# ABAG-docking benchmark: a non-redundant structure benchmark dataset for antibody–antigen computational docking

**DOI:** 10.1093/bib/bbae048

**Published:** 2024-02-21

**Authors:** Nan Zhao, Bingqing Han, Cuicui Zhao, Jinbo Xu, Xinqi Gong

**Affiliations:** Institute for Mathematical Sciences, School of Mathematics, Renmin University of China, Beijing, China; Institute for Mathematical Sciences, School of Mathematics, Renmin University of China, Beijing, China; Institute for Mathematical Sciences, School of Mathematics, Renmin University of China, Beijing, China; MoleculeMind Ltd., Beijing, China; Institute for Mathematical Sciences, School of Mathematics, Renmin University of China, Beijing, China; Beijing Academy of Artificial Intelligence, Beijing, China

**Keywords:** docking benchmark, antibody–antigen complex, docking method, structure prediction

## Abstract

Accurate prediction of antibody–antigen complex structures is pivotal in drug discovery, vaccine design and disease treatment and can facilitate the development of more effective therapies and diagnostics. In this work, we first review the antibody–antigen docking (ABAG-docking) datasets. Then, we present the creation and characterization of a comprehensive benchmark dataset of antibody–antigen complexes. We categorize the dataset based on docking difficulty, interface properties and structural characteristics, to provide a diverse set of cases for rigorous evaluation. Compared with Docking Benchmark 5.5, we have added 112 cases, including 14 single-domain antibody (sdAb) cases and 98 monoclonal antibody (mAb) cases, and also increased the proportion of Difficult cases. Our dataset contains diverse cases, including human/humanized antibodies, sdAbs, rodent antibodies and other types, opening the door to better algorithm development. Furthermore, we provide details on the process of building the benchmark dataset and introduce a pipeline for periodic updates to keep it up to date. We also utilize multiple complex prediction methods including ZDOCK, ClusPro, HDOCK and AlphaFold-Multimer for testing and analyzing this dataset. This benchmark serves as a valuable resource for evaluating and advancing docking computational methods in the analysis of antibody–antigen interaction, enabling researchers to develop more accurate and effective tools for predicting and designing antibody–antigen complexes. The non-redundant ABAG-docking structure benchmark dataset is available at https://github.com/Zhaonan99/Antibody-antigen-complex-structure-benchmark-dataset.

## INTRODUCTION

Proteins mostly interact with other proteins to form complexes that perform biological functions [1]. Accurately identifying protein–protein interactions (PPIs) helps to gain insight into the functions of unknown proteins, reveal the biological mechanisms of life activities or diseases and thus promote drug design research [2]. Antibody–antigen interaction is a well-characterized class of PPIs, which is an essential part of the immune response of organisms to pathogens [3]. Understanding the structural basis of antibody–antigen interactions can help design more effective therapeutics. Experimental structure characterization methods such as X-ray crystallography, nuclear magnetic resonance and cryo-electron microscopy are more accurate methods and can provide significant information on key residues involved in antibody–antigen interactions [4, 5]. However, the experimental methods have the disadvantages of being time-consuming, costly and labor-intensive, especially the large number of immune repertoires and antigen targets increase the technical difficulty and cost of experimental structure characterization [6]. Consequently, various computational approaches have been developed to provide valuable and fast alternatives to experimental techniques.

Many protein–protein docking methods have been utilized to predict PPIs and protein complex structures. Traditional docking methods generally use search strategies to sample a large number of candidate conformations and then use scoring functions to rerank and select the generated conformations. The main difference among these methods is the use of different search strategies to optimize the calculation. It mainly includes the following three categories: (i) fast Fourier transform (FFT) like ZDOCK [7–9] and ClusPro [10, 11]; (ii) local shape feature matching method like PatchDock [12] and (iii) random search like Rosetta [13–15], ATTRACT [16] and HADDOCK [17] using Monte Carlo search. Several of these methods have specific modes for antibody–antigen docking (ABAG-docking), such as ClusPro [10], FRODOCK [18, 19], PatchDock [12], HADDOCK [17] and Rosetta SnugDock [20]. When the binding information is available, the search space for docking can be greatly reduced with the help of epitope or paratope information. Deep learning technologies are developing rapidly, and they are also used in docking algorithms and complex structure prediction. The deep learning–based docking algorithm avoids the search process and improves the running speed compared with traditional docking methods. They utilize graph matching networks, invariant point attention and other techniques for docking, such as EquiDock [21], DockGPT [22] and GeoDock [23]. AlphaFold2 [24] has achieved great success and is an end-to-end deep learning algorithm that can predict protein monomer structures based on the amino acid sequence, multiple sequence alignment (MSA) and homologous structure information. Subsequently, based on AlphaFold2, AlphaFold-Multimer [25] was developed for protein complex structure prediction. Although many computational methods have been developed, it is common for molecular docking to be inaccurate, especially when dealing with flexible molecules such as antibodies. There is still a need to improve the ABAG-docking algorithms.

The development of ABAG-docking computational methods relies on benchmark datasets. The structure of the antibody–antigen interaction has been published in a public repository. Large-scale curation efforts have resulted in databases of well over a billion antibody sequences without a target or binding affinity values. Other efforts have resulted in datasets with close to 1000 antibodies with labels—either target sequences or neutralization values. There are 40 cases of antibody–antigen complexes in Docking Benchmark 5.0 (BM5) [6] and 67 cases in BM5.5 [26]. The small dataset of antibody–antigen complex structures makes assessments specific for ABAG-docking somewhat difficult.

In this work, we first reviewed previously developed ABAG-docking datasets, including Benchmark versions 4.0, 5.0 and 5.5, in terms of dataset sizes, composition, etc., and pointed out areas for improvement. Furthermore, we developed a non-redundant ABAG-docking benchmark of 112 cases for antibody–antigen complex structures after conducting a comprehensive search of the Protein Data Bank (PDB) [27] and other databases including the Summary of Antibody Crystal Structures (SACS) [28] and Structural Antibody Database (SAbDab) [29]. Each case in the benchmark dataset includes a bound antibody–antigen complex structure and the unbound structures of its antibody and antigen components. The unbound structure was obtained from monomers, dimers or other complexes. These cases supplemented 67 antibody–antigen complex cases from BM5.5, increasing the total number of cases to 179. Both antibody and antigen structures are flexible and diverse, so we also collected different antibody–antigen conformations and analyzed their conformational changes. We collected as many different unbound structures of the antigen/antibody in the complex as possible and retained antibodies and antigens of various lengths, including peptide antibodies and peptide antigens. We also recorded both unbound–bound and bound–unbound cases. The analysis of the success rate of various complex prediction algorithms conducted on the collected ABAG-docking benchmark complexes reveals the problems and challenges in the current docking algorithms and benchmark datasets. This benchmark dataset will facilitate the development and improvement of protein docking and sequence-based protein complex structure prediction algorithms. This benchmark dataset is available at https://github.com/Zhaonan99/Antibody-antigen-complex-structure-benchmark-dataset.

### Overviews of ABAG-docking datasets

The previous version of the Docking Benchmark 4.0 (BM4) [30] included 175 cases, which were further categorized into various binding modes across different cases. BM5 significantly expanded the dataset to 230 cases, representing a 31.42% increase from the previous 175 cases. These protein complexes are typically classified into different docking difficulty levels, including ‘Rigid’, ‘Medium’ and ‘Difficult’. Table 1 illustrated the composition of the updated docking benchmarks. In BM4, there were 119 Rigid cases, 29 Medium cases and 27 Difficult cases. With the expansion in BM5, the number of Rigid cases increased to 151 (a 26.89% increase), the number of Medium cases rose to 45 (a 55.17% increase) and the number of Difficult cases reached 34 (a 25.93% increase). Furthermore, with the expansion in BM5 to BM5.5 [26], the number of Rigid cases increased to 162 (a 7.28% increase), the number of Medium cases rose to 60 (a 33.33% increase) and the number of Difficult cases reached 35 (a 2.94% increase). Notably, in terms of antibody–antigen interactions, BM4 contained only 24 antibody–antigen complexes, accounting for a mere 13.71% of the entire dataset. In BM5, the number of antibody–antigen complex structures was expanded from the initial 24 to 40, now representing 17.39% of the dataset in the current version. In BM5.5, the number of antibody–antigen complex structures has been expanded from 40 to 67, accounting for 26.07% of the current version of the dataset. As can be seen, such data are gradually increasing, reflecting the growing interest in antibody-based therapies.

**Table 1:** Composition of the updated docking benchmarks

	**Benchmark version 4.0**	**Benchmark version 5.0**	**Benchmark version 5.5**
All	175	230	257
Rigid	119	151	162
Medium	29	45	60
Difficult	27	34	35
Antibody–antigen	24	40	67
Others	151	190	190

Due to the limited number of antibody–antigen complex datasets, BM5.5 was introduced in 2021 with a focus on improving the evaluation of antibody–antigen complex structures. Specifically, this version expanded the dataset from 40 cases in version 5.0 to 67 cases. Table 2 presents the structural composition of antibody–antigen complexes with different docking benchmarks. When concentrating on the docking difficulty levels of antibody–antigen data, it becomes evident that previous benchmarks (BM4 and BM5) contained very few Medium and Difficult cases, accounting for only 8.33 and 17.50% of the total dataset, respectively. This skewed the evaluation of docking methods. In BM5.5, this issue was addressed by expanding to 23 Medium and Difficult antibody–antigen complexes, making up 34.33% of the total dataset. However, these cases still represent a minority compared with Rigid cases. Moreover, redundant antibody–antigen examples with a single ID corresponding to multiple cases were removed during the removing redundancy process in BM5 and BM5.5. Additionally, when considering the statistics of antibody types, it is evident that the number of mAbs significantly outweighs the number of sdAbs in each version.

**Table 2:** Antibody-antigen complex structure composition of the different docking benchmarks

	**Benchmark version 4.0**	**Benchmark version 5.0**	**Benchmark version 5.5**
All antibody-antigen cases	24	40	67
Rigid	22	33	44
Medium or Difficult	2	7	23
Cases with distinct binding modes	1 ID: 1QFW	0	0
mAb	22	39	54
sdAb	2	2	13

ABAG-docking datasets still have fewer cases and are lacking Medium or Difficult cases as well as cases with two interfaces. Imperfections in datasets pose significant challenges to the development of docking algorithms.

## MATERIALS AND METHODS

### Benchmark construction summary

In the data construction phase, three sources were utilized to ensure comprehensive coverage and obtain a large number of antibody–antigen complexes. We obtained two lists of antibody-containing structures from the SACS [28] resource and the SAbDab [29] resource. Furthermore, we also identified antibody–antigen complexes by an advanced search in the PDB resource. While the SACS and SAbDab databases already contain data from the PDB, our approach is driven by the fact that our initial PDB searches rely on keywords like ‘antibody’ and ‘complex’, which could potentially overlook certain complexes. However, any antibody–antigen complexes that are missed via advanced search in the PDB can be located and added to our dataset from the other two databases for supplementation. Our analysis revealed that the antibody–antigen complexes sourced from the three resources are complementary, and no resource can fully cover them. Complexes were only released after 1 June 2019 (based on the BM5.5 dataset update in May 2019), and structures that only contained antibodies were not included in our dataset. To identify the unbound structures of antibodies and antigens within each antibody–antigen complex, we performed the BLAST [31, 32] program to search against all amino acid sequences in the PDB. Various criteria were used to identify the unbound structures:


(1) \begin{equation*} Identity > 93\%, Alignment\ coverange > 80\%, E- value < {10}^{-5} \end{equation*}

To ensure a fair evaluation of the docking accuracy, we have performed the de-redundancy on the dataset. Two structures are considered redundant if the sequence alignments evaluated by Identity and E-value [Equation (2)], and structure alignment results evaluated by root-mean-square distance (RMSD) [Equation (3)] satisfy


(2) \begin{equation*} Identity > 60\%,\ E- value<{10}^{-30} \end{equation*}

(3) \begin{equation*} RMSD < 5 \overset{\circ}{\text A} \end{equation*}

**Figure 1:**
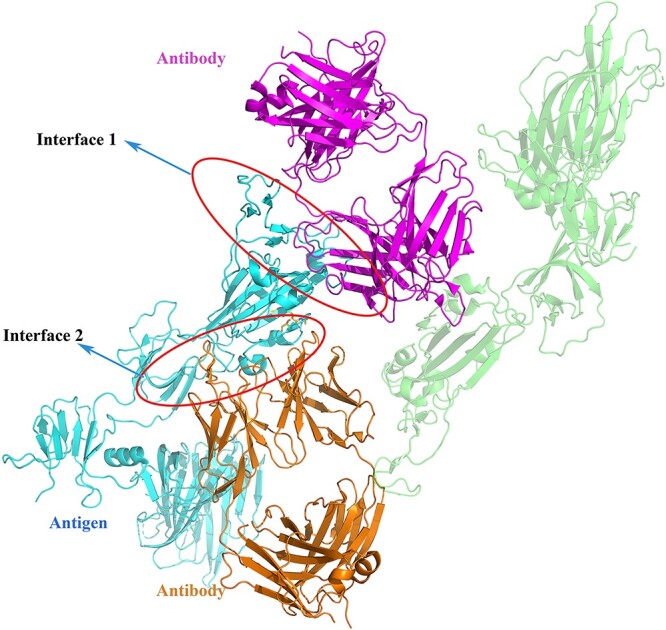
An illustration of the case with two antibody–antigen interfaces. Complex 7A5S has two distinct interfaces: interface 1 and interface 2, which are highlighted in circles.

After the de-redundancy stage, 105 antibody–antigen complexes were screened that contained both unbound antibodies and antigens structures. We prepared structure files for the bound and unbound structures of the above complexes as a benchmark dataset. Similar to the data processing in BM5, each complex in the dataset contains the fewest protein chains that correctly reflect the binding process [6]. For the structure files, we aligned the bound and unbound structures and kept only the ATOM fields. For some complexes containing two antibody–antigen interfaces, we also prepared two sets of structure files accordingly. As shown in Figure 1, complex 7A5S has two interfaces. Comparing the two interfaces, the antibody and antigen components have different binding sites. Some unbound sequences are much longer than bound sequences. For these cases, we truncated the unbound sequences to match the lengths of the bound sequences, leaving only a portion of the unbound structure to facilitate docking. For convenience of use, we also provided untruncated PDB format files for the 25 truncated cases.

### Docking difficulty classification

We analyzed conformational changes and docking difficulty by calculating several properties of the structure files. We calculated the change in solvent-accessible surface area (∆ASA) during binding by using the NACCESS [33] algorithm. We also estimated the docking difficulty of the cases by utilizing the interface RMSD (I-RMSD) and the fraction of non-native contacts ${f}_{non- nat}$ between bound and unbound structures after the optimal superimposition of the interface. Among them, I-RMSD is the ${C}_{\alpha }- RMSD$ (that is, using ${C}_{\alpha }$ atoms to calculate the RMSD) of interface residues, which is calculated by aligning the unbound structure to the bound structure. In the calculation of I-RMSD, the interface residues are defined as those within 10 Å of any non-hydrogen protein atoms in the binding partner. The contact definition of ${f}_{non- nat}$ is similar to that of the I-RMSD, but the threshold is 5 Å.

**Figure 2:**
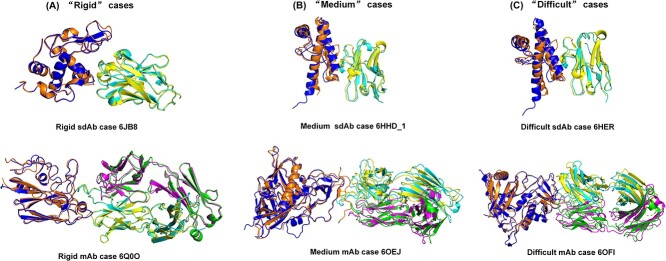
Several representative cases for three difficulty levels: Rigid, Medium and Difficult. (**A**) Rigid case: sdAb case 6JB8($\mathrm{I}-\mathrm{RMSD}=0.938 \overset{\circ}{\text A},{f}_{non- nat}=0.344$) and mAb case 6Q0O ($\mathrm{I}-\mathrm{RMSD}=0.574 \overset{\circ}{\text A},{f}_{non- nat}=0.141$); (**B**) Medium case: sdAb case 6HHD_1 ($\mathrm{I}-\mathrm{RMSD}=2.153 \overset{\circ}{\text A},{f}_{non- nat}=0.545$) and mAb case 6OEJ ($\mathrm{I}-\mathrm{RMSD}=0.965 \overset{\circ}{\text A},{f}_{non- nat}=1.000$) and (**C**) Difficult case: sdAb case 6HER ($\mathrm{I}-\mathrm{RMSD}=2.354 \overset{\circ}{\text A},{f}_{non- nat}=0.583$) and mAb case 6OFI ($\mathrm{I}-\mathrm{RMSD}=2.956 \overset{\circ}{\text A},{f}_{non- nat}=0.661$).

We classify 112 cases into three categories: ‘Rigid’, ‘Medium’ and ‘Difficult’. The statistical data and criteria for classification [26] with different properties are presented in Table 3. It can be observed that the dataset comprises two-thirds of Medium and Difficult cases. These three categories of cases represent the challenges encountered in docking. Therefore, the dataset we’ve collected can complement BM5 and BM5.5 datasets, ensuring a more balanced representation of all three difficulty levels. Furthermore, Table 3 displays the count of cases associated with different antibody types in each case. And Figure 2 shows representative cases falling into the ‘Rigid’, ‘Medium’ and ‘Difficult’ categories. As seen in Figure 2, the Rigid cases, such as 6JB8 and 6Q0O, exhibit minimal conformational changes, with nearly identical backbones between the bound and unbound structures. In contrast, the Medium cases, like 6HHD_1 and 6OEJ, display significant conformational alterations at the interface. For the Difficult cases, such as 6HER and 6OFI, considerable conformational changes occur, especially at the interface between the bound and unbound structures, resulting in substantial non-overlap.

**Table 3:** The statistical data and criteria for classifying cases by $\mathrm{I}-\mathrm{RMSD}$ and ${f}_{non- nat}$

**Category**	**Criterion**	**Number of cases**	**Antibody Type: Number**
Rigid	$\mathrm{I}-\mathrm{RMSD}<1.5 \overset{\circ}{\text A},{f}_{non- nat}<0.40$	31	sdAb: 8
			mAb: 23
Difficult	$\mathrm{I}-\mathrm{RMSD}>2.2\ \overset{\circ}{\text A}$	36	sdAb: 4
			mAb: 32
Medium	other	45	sdAb: 2
			mAb: 43

### Curating diverse types of data

**Figure 3:**
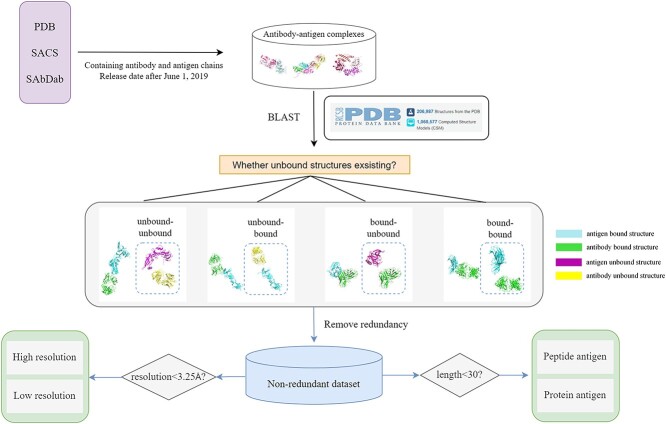
Dataset classification. The antibody and antigen components of the antibody–antigen complexes were individually aligned with sequences in PDB. Based on the availability of corresponding unbound structures for the antibody/antigen's bound configurations within the comparative analysis, these complexes can be categorized into four distinct groups: unbound-unbound, unbound-bound, bound-unbound, and bound-bound. Non-redundant datasets can be further divided based on resolution and antigen length.

Due to the limited number of antibody–antigen complex structures in the PDB, we have retained a list of all antibody–antigen complexes obtained from the three mentioned resources. To cater to the diverse needs of various tasks, we have divided the dataset based on different criteria. For the antibody–antigen complexes obtained from PDB, SACS and SAbDab, we used the BLAST program to search for their unbound structures in the PDB database (Figure 3). Based on the existence of unbound structures, these complexes are further divided into unbound–unbound, unbound–bound, bound–unbound and bound–bound cases. Among them, the unbound–unbound case is the complex in which both antibody and antigen have unbound structures. The unbound–bound case is the complex in which the antibody has unbound structures but the antigen does not. The bound–unbound case is the complex in which the antibody does not have unbound structures but the antigen has unbound structures. The bound–bound case is the complex in which neither antibody nor antigen has unbound structures. We remove redundant complexes from the dataset to obtain a non-redundant dataset. In addition, we also annotated the lengths of antigen chains and the resolution information. Based on the lengths of antigens, data can be divided into complexes that contain peptide antigens and protein antigens. According to resolution, data can be categorized into high-resolution complexes and low-resolution complexes.

### Generating ABAG-docking benchmark dataset

**Figure 4:**
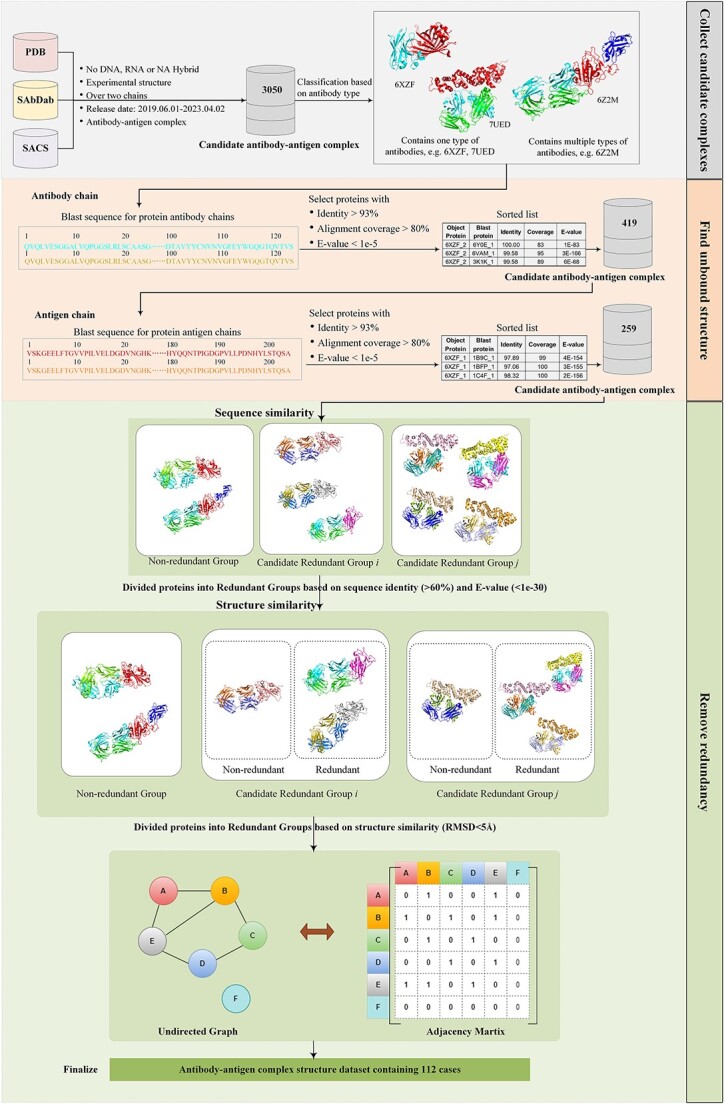
Pipeline for constructing antibody–antigen complex dataset.

The different stages for the ABAG-docking benchmark dataset are shown in Figure 4. The process consists of three steps: collecting candidate antibody–antigen complexes, finding unbound structures of antibody chains and antigen chains and removing redundancy. The steps are described below:

#### Collecting candidate antibody–antigen complex

We collected candidate antibody–antigen complexes from PDB, SAbDab and SACS databases. After we restricted the dates to after 1 June 2019, and filtered out DNA, RNA and NA-hybrid experimental structures, a total of 3050 antibody–antigen complexes were selected for further analysis. On the PDB website, each complex contains a list of features. Among those, these are 10 features selected as they are more commonly used for antibody–antigen complex structure analysis: PDB ID, Experimental Method, Release Date, Resolution, Sequence, Entity Polymer Type, Polymer Entity Sequence Length, Source Organism, Macromolecule Name and Chain ID.

#### Finding unbound structures

In order to identify the unbound structures, the process involves comparing the antibody and antigen chains within potential antibody–antigen complexes to the full amino acid sequences. Initially, the unbound structure of the antibody chain is sought. The antibody chains in the candidate antibody–antigen complexes are separated and aligned with the full amino acid sequence database in the PDB library by utilizing BLAST. Then, a similar procedure is employed to conduct an unbound structure search for the antigen chains within the antibody–antigen complexes. The resulting dataset contains proteins in which both the antibody and antigen have unbound structures, and these are retained as the newly filtered candidate antibody–antigen complex data.

#### Analyzing sequence and structural similarities

**Figure 5:**
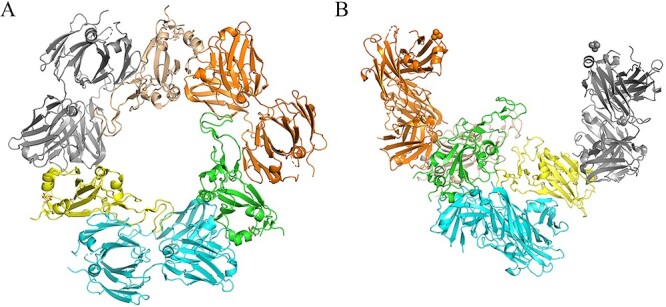
Cases with similar sequences but distinct structures. (**A**) Structure of complex 6ZDG. (**B**) Structure of complex 6ZER. Despite having highly similar sequences (100% identity in all three chain types), these two complexes exhibit markedly different structures.

Highly similar sequences usually belong to homologous sequences that are more likely to have similar structures and functions. Therefore, when calculating sequence similarity between different protein complexes, sequences are considered redundant if sequence identity is >60% and the e-value is <${10}^{-30}$. However, some proteins, despite having highly similar amino acid sequences, do not always share similar three-dimensional structures. For example, protein 6ZDG and protein 6ZER have up to 100% sequence identity for the three kinds of chains, but their structures are relatively different (Figure 5). Therefore, two proteins with high sequence identity are not necessarily redundant; they may have different structures, and further research on their structural similarity is investigated to make a judge. To compare the structural similarity of two proteins within the same sequence redundant group, we aligned the complex structures and calculated the RMSD value, which indicates the structural similarity between the two antibody–antigen complexes. If the RMSD <5 Å and Equation (2) is satisfied, these two protein complexes are deemed redundant.

#### Removing redundancy

**Figure 6:**
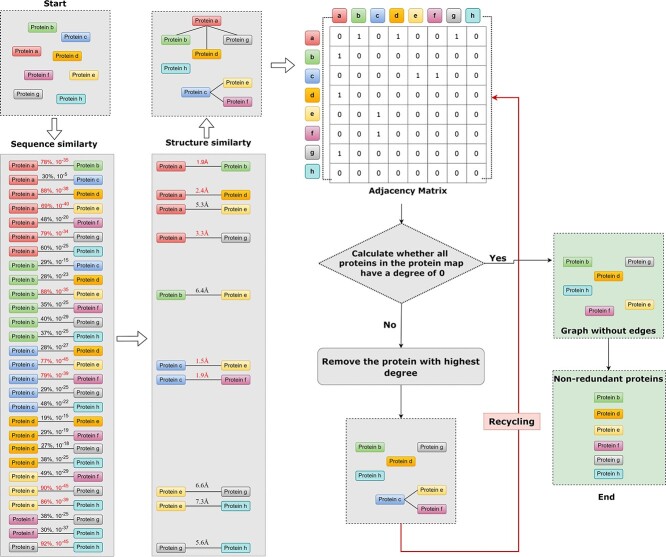
A pipeline for removing redundant.

We considered each protein to be a node and only established an edge between two proteins if they were considered redundant; otherwise, no connection was established. This method can lead to the creation of an undirected graph. To begin, we calculated the adjacency matrix of this undirected graph and the degree of each node. Subsequently, we arranged these degrees in descending order, identifying the protein with the highest degree as the next one to be removed. If multiple proteins shared the maximum degree, we randomly chose one for deletion. We then eliminated the target protein along with its connected edges, giving rise to a new undirected graph. We recalculated the adjacency matrix and node degrees for this new graph, removed the node with the maximum degree and its adjacent edges and repeated this process until the degree of each node reached 0. At this stage, there were no connected edges between any nodes in the graph, which indicated no two proteins were considered redundant. This method effectively transformed the redundant group into a non-redundant one. The specific pipeline for removing redundancy is shown in Figure 6.

### Updating ABAG-docking benchmark dataset

**Figure 7:**
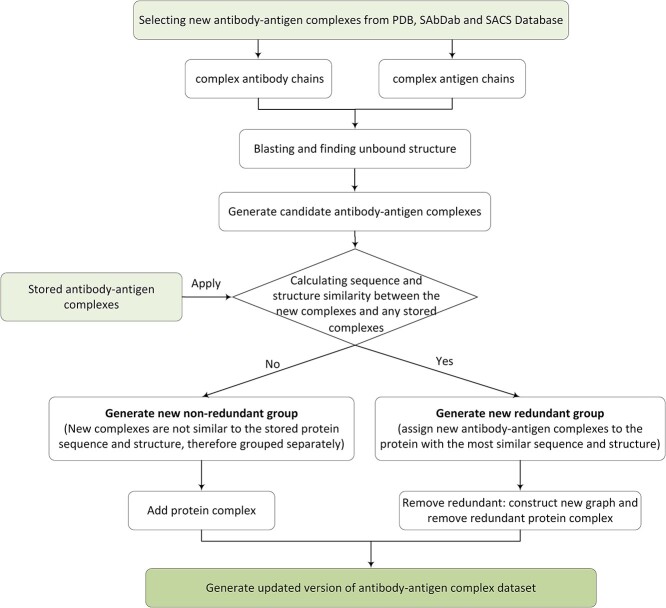
A pipeline for updating the antibody–antigen complex structure dataset.

The PDB database is a high-growth database, with statistics from the past few decades showing an average release of 525 new antibody-related structures per year. The SAbDab and SACS databases contain antibody structures that are available in the PDB. To incorporate the newly introduced antibody–antigen complexes into the non-redundant dataset, it is crucial to regularly update the antibody–antigen complex dataset. Collecting this dataset from scratch for filtering and removing redundancy each time is a laborious and complex process. Therefore, we have devised a pipeline for updating the dataset by comparing the newly added antibody–antigen complexes with those in the ABAG-docking benchmark. Figure 7 provides an overview of the key steps involved in the antibody–antigen complex update pipeline, which will be briefly discussed in this section.

#### Selecting new antibody–antigen complexes

The newly added antibody–antigen complexes in PDB, SAbDab and SACS databases and their important features were collected with limited release dates and periods, excluding complexes with DNA, RNA and NA Hybrid chains.

#### Finding unbound structures and generating candidate complexes

The complexes obtained in the previous step are grouped into antibody chains and antigen chains, and their sequence alignments with the full amino acid sequences in PDB are performed by BLAST, respectively. The selection of candidate antibody–antigen complexes, as well as their antibody and antigen unbound structures, is made based on the alignment results of sequence identity >93%, alignment coverage >80% and $E- value<{10}^{-5}$.

#### Calculating the similarity between new and stored complexes

The newly selected antibody–antigen complexes are assessed by comparing them to the complexes stored in the previous version of the antibody–antigen complex dataset. To be classified as similar, a new antibody–antigen complex must satisfy certain criteria when compared with a stored complex: sequence identity >60%, sequence $E- value<{10}^{-30}$ and structure RMSD <5 Å.

#### Generating a new non-redundant group

When assessing a new antibody–antigen complex, if it fails to meet the sequence and structural similarity criteria when compared with all previously stored complexes, it is deemed that the new antibody–antigen complex is distinct from any of the stored ones. Consequently, it establishes a distinct, non-redundant group. This newly identified complex is then included in the updated dataset, which combines the stored complexes with the newly added complex. The updated dataset can be regarded as an enhanced version of the non-redundant antibody–antigen complex dataset.

#### Generating a new redundant group

When dealing with a new antibody–antigen complex, we assess its compatibility with the previously stored complexes by examining both sequence and structural similarities. If the new complex exhibits substantial similarity to any of the stored ones, it is deemed a match, leading to the creation of a new redundant group. In such cases, a new graph is created, incorporating all previously stored complexes and newly collected ones. Subsequently, node degrees are calculated, and the redundant protein complexes are eliminated to ensure the removal of redundancy.

## RESULTS

### Benchmark composition

**Figure 8:**
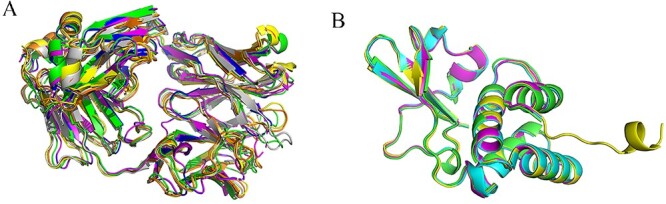
The antibody and antigen components of complex 6OTC and their unbound structures. (**A**) The antibody component of 6OTC and its unbound structures. According to the sequence alignment results, the antibody component of 6OTC has multiple unbound structures and shows different conformational changes (5VZ1: RMSD = 0.790 Å; 6B6Z: RMSD = 0.784 Å; 7T98: RMSD = 1.975 Å; 7VMZ: RMSD = 2.127 Å; 7T99: RMSD = 2.254 Å). (**B**) The antigen component of 6OTC and its unbound structures. The antigen component of 6OTC also has multiple unbound structures (4GH9: RMSD = 0.538 Å; 4GHA: RMSD = 0.416 Å; 4GHL: RMSD = 0.431 Å)

From the above three resources, we obtained a total of 3050 antibody–antigen complex structures that were released after May 2019. We searched unbound structures for antibody and antigen chains of these complexes and obtained a total of 259 initial unbound–unbound cases. A total of 112 non-redundant unbound–unbound cases were obtained after removing redundancy, which are referred to as the ABAG-docking benchmark dataset (summarized in Supplementary Excel1). In addition, we also provided unbound–bound, bound–unbound and bound–bound cases to expand the dataset for different needs such as predicting antibody–antigen complexes from sequences and docking using modeled unbound antibody structures. Specifically, all four kinds of cases can be used to test sequence-based antibody–antigen complex prediction methods. The bound-unbound example can be utilized to evaluate the performance of docking using modeled antibody structures. After removing redundancy, the bound-unbound type has a total of 865 cases (in Supplementary Excel2 sheet ‘bound-unbound’); the unbound–bound type has a total of 99 cases (in Supplementary Excel2 sheet ‘unbound-bound’). For each case, we collected as many unbound structures as possible. Multiple unbound structures may exist within the antibody and antigen components of the antibody–antigen complexes, where some have small conformational changes and others have large conformational changes. During docking, it may be necessary to start from different unbound structures to generate complexes as similar to the native conformation as possible. For example, Figure 8 shows the diversity of the unbound structures of the antibody component and antigen component of complex 6OTC, respectively. We collected various unbound structures of three types of cases including unbound–unbound, unbound–bound and bound–unbound and put their corresponding IDs in Supplementary Excel2.

Based on the calculated I-RMSD and ${f}_{non- nat}$, 27.68% of the cases were classified as Rigid, 40.18% of the cases were classified as Medium and 32.14% of the cases were classified as Difficult. Our dataset is enriched with cases at Medium or Difficult levels, posing challenges for more complete solutions to the ABAG-docking problem. 57.14% of sdAb cases are classified as Rigid cases, while 23.47% of mAb cases are classified as Rigid cases. In comparison, the sdAb cases are easier to dock.

**Figure 9:**
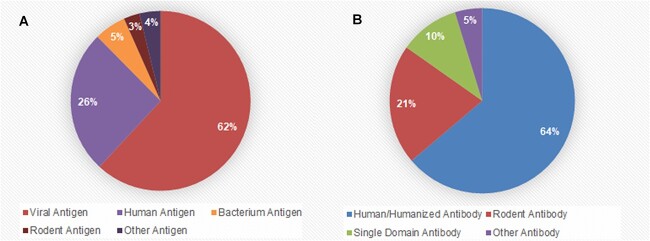
Species sources composition of antibody chains and antigen chains in the ABAG-docking benchmark dataset. (**A**) The species source composition of antibody chains. The largest source of antibody chains is human/humanized antibody, accounting for 64%. (**B**) The species source composition of antigen chains. The largest source of antigen chains is viral antigens, accounting for 62%.

The antibodies in the non-redundant unbound–unbound dataset can be further categorized based on their sources (Table S5), including human/humanized antibodies, single-domain antibodies, rodent antibodies and other types (Figure 9A). Similarly, antigens in the dataset can be categorized based on their sources, including virus antigens, *Homo sapiens* antigens, bacterium antigens, rodent antigens and other types (Figure 9B). The virus antigens category encompasses a range of specific antigens like HIV-1 CLADE A/E GP120, MERS-CoV and SARS-CoV-2, among others.

**Figure 10:**
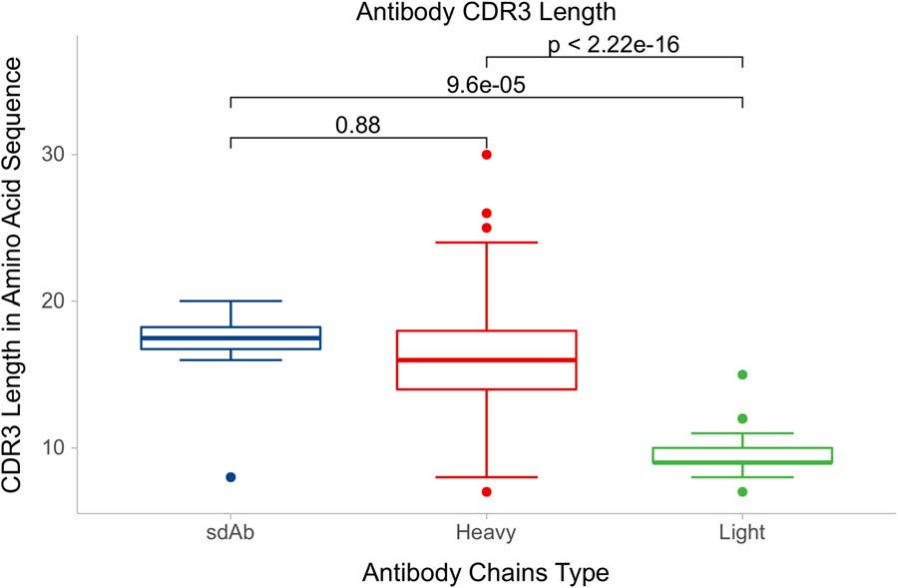
Box plot of the CDR3 lengths for sdAb, mAb heavy chains and mAb light chains. A significant distinction in CDR3 lengths is observed between the mAb light chain group (*n* = 92) and the sdAb group (*n* = 13), as indicated by the *t*-test results.

In addition, we recorded the lengths of the antigen chains, which ranged from 53 to 1288 residues (Table S2). Considering that there will be more data in the future, these data are necessary, and the cases can be further divided into peptide antigen cases and protein antigen cases. We also recorded the resolution and the release date of the cases (Table S3), so that users can choose a subset of the dataset according to their own needs. We conducted a comprehensive analysis of complementarity determining region (CDR)3 lengths in antibodies across all antibody–antigen complexes. We found that the CDR3 lengths of mAb cases range from 7 to 30 residues, whereas the CDR3 lengths of sdAb cases range from 8 to 20 residues (Table S4). In these cases, CDR3 regions have a significant variation in their sequence lengths. The benchmark dataset shows that the CDR3 lengths of sdAbs have a noticeably longer average length than the CDRL3 lengths of mAbs (as depicted in Figure 10).

### Binding conformational changes

We calculated the binding conformational changes of the non-redundant ABAG-docking benchmark from multiple perspectives referring to BM5.5, analyzed the patterns of conformational changes and faced challenges in docking calculations.

#### Overall changes

**Figure 11:**
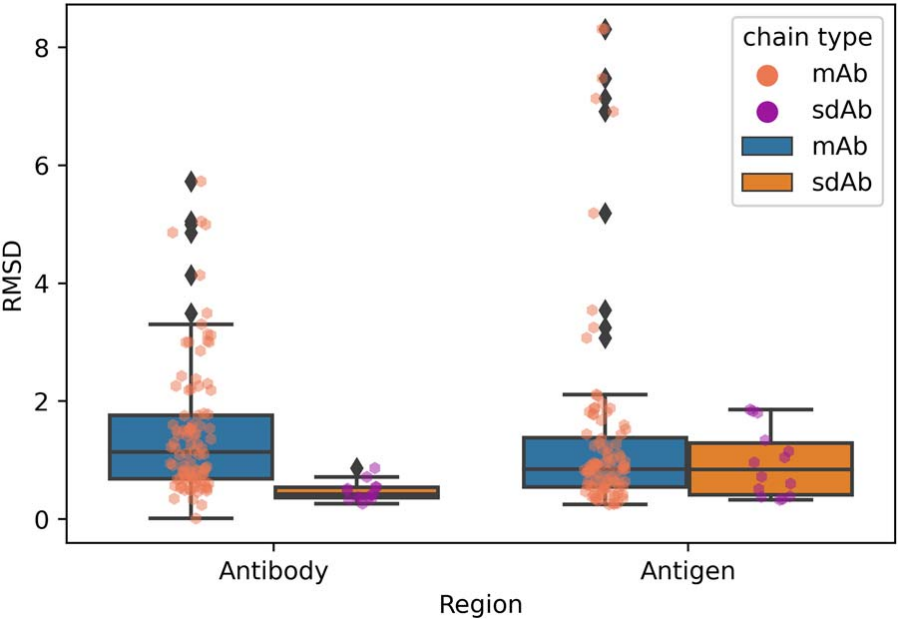
RMSDs of antibody and antigen components. For clarity, RMSD values are limited at 20 Å in this plot, and two cases with antigen RMSD >20 Å are not shown. These values occur all in antigen components (6LGW: 20.33 Å; 7KDD: 23.73 Å).

We calculated the RMSDs for unbound and bound antibody structures as well as for antigen structures, respectively. Figure 11 shows the structural changes involved in antigen and antibody binding. Overall, the antibodies and antigens remained relatively unchanged after binding, with median RMSDs <1.2 Å and RMSD <3 Å in most cases. Figure 11 shows that complexes containing mAb have a wider range of RMSDs between unbound and bound structures, and the median of mAb antibody RMSDs is significantly higher than the median of sdAb antibody RMSDs (*P*-value = 1.53e-06 determined by Wilcoxon rank-sum test). The sdAb cases are easier to dock than the mAb cases.

#### I-RMSD calculation

We calculated the I-RMSDs for antibody as well as for antigen structures separately and the entire interface I-RMSDs. Although most cases have I-RMSD <4 Å, remaining relatively static before and after binding, the I-RMSDs’ range is widely distributed (0–20 Å), which will bring some challenges to ABAG-docking.

**Figure 12:**
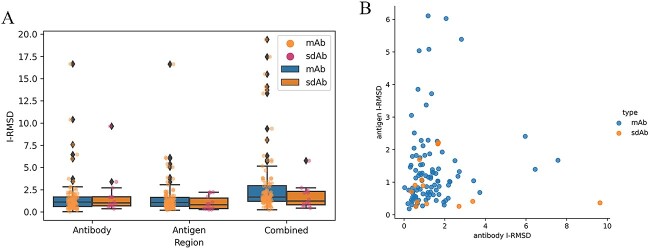
I-RMSDs of cases. (**A**) I-RMSDs of antibody, antigen and entire interface. (**B**) Antigen versus antibody I-RMSD. For clarity, I-RMSD values are limited at 10 Å in (**B**), and two cases with antibody I-RMSD >10 Å (7WRV: 10.38 Å; 7YQZ_2: 16.64 Å) and one case with antigen I-RMSD >10 Å (7KDD: 16.61 Å) are not shown.

The antigen and combined I-RMSDs of mAb chains are higher than those of sdAb chains, but the antibody I-RMSDs of sdAb and mAb are at the same level. The I-RMSDs of mAb have a larger range of variation than that of sdAb (Figure 12A). As shown in Figure 12B, if the antibody or antigen I-RMSD is large, the I-RMSD on the other side of the interface will be relatively low. This may be because the overall interface conformation changes are limited (the I-RMSDs for the entire interface of most cases are lower than 6 Å. When one side of the interface changes high, the other side of the interface changes relatively low due to this limitation). The I-RMSD range of sdAb cases is smaller than that of mAb cases, so it is important to consider a wider range of conformational changes for ABAG-docking involving mAb.

#### CDR RMSD calculation

**Figure 13:**
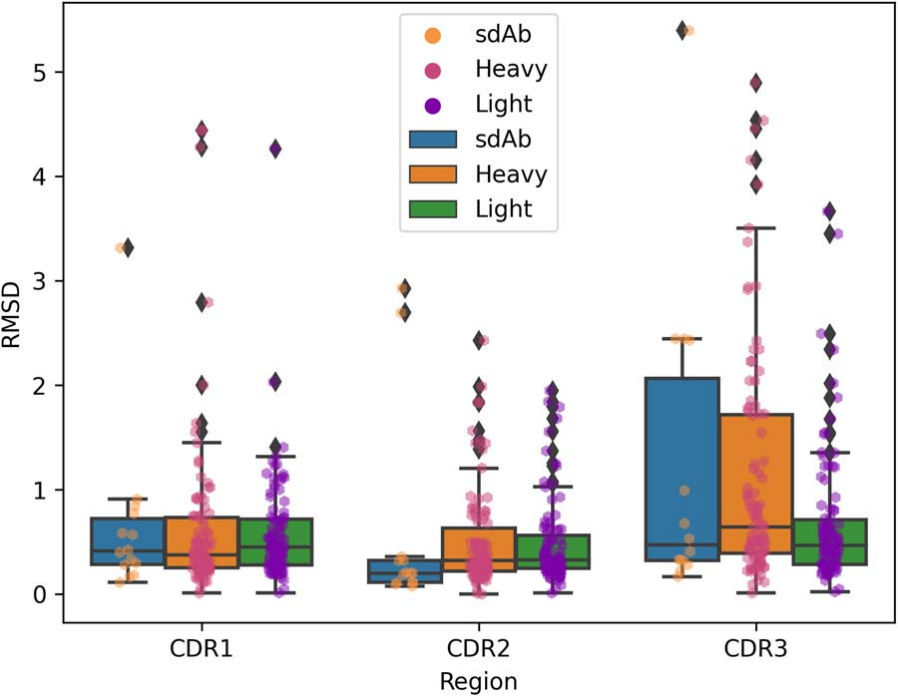
RMSDs of CDR loops. CDR definitions are based on Chothia numbering.

The CDR is the hypervariable region of the antibody that is prone to conformational changes. For these regions, we also calculated the RMSDs separately. Figure 13 illustrates a wider range of conformational changes in CDR3 compared with CDR1 and CDR2, showcasing some significant conformational changes (>4 Å). In the CDR1 region, the RMSDs for the sdAb chain, light chain and heavy chain exhibit no significant differences. However, in the CDR2 regions, the RMSDs for the heavy chain and light chain are notably higher than those for sdAb chains (*P*-value = 0.034 and *P*-value = 0.018, respectively). Notably, in the CDR3 regions, the CDRs of heavy chain are significantly higher than light chains (*P*-value = 0.003).

### Antibody–antigen complex structure prediction

**Figure 14:**
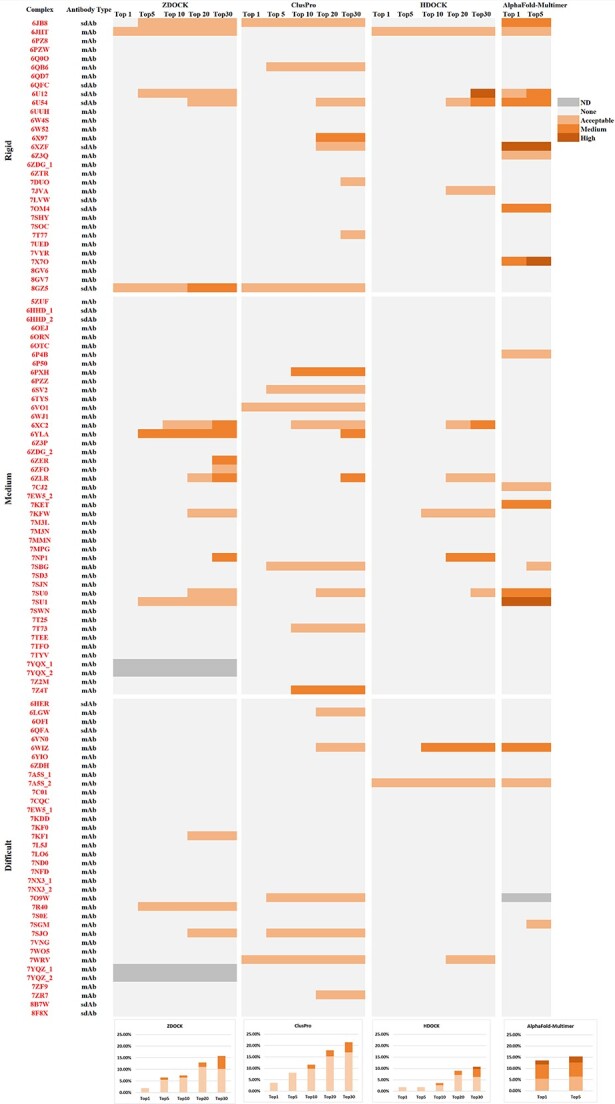
Antibody–antigen complex prediction performance on ABAG-docking benchmark. In the figure, ‘ND’ stands for ‘no docking result of target’. For ZDOCK, cases 7YQX_1, 7YQX_2, 7YQZ_1, 7YQZ_2 are too large to be docked. For AlphaFold-Multimer, case 7O9W has no prediction result due to our device limitation.

The result analysis of a docking benchmark test helps to objectively evaluate the performance of the docking algorithms. Through prediction results, especially failed predictions, users can compare the performance differences of existing complex prediction algorithms and summarize the advantages and disadvantages of different complex prediction algorithms. Researchers can evaluate the accuracy and reliability of these methods by comparing predicted structures with experimental complexes. Thus, users can provide more useful insights into existing problems and further improve and develop new complex prediction methods. To preliminarily assessment the differences between test cases and their docking difficulty, we used three global docking algorithms: ZDOCK [7, 9], ClusPro [10] and HDOCK [34, 35]. We used the unbound structures in the collected antibody–antigen complex dataset as input to conduct global protein docking simulations. We also used the amino acid sequences of the antibody–antigen complexes as input for folding predictions by AlphaFold-Multimer (Figure 14). And we evaluated the prediction results by DockQ [36]. We listed the details of the dataset and docking algorithm (Table S1).

#### ZDOCK

ZDOCK is a docking algorithm that uses the FFT [37] for rapid rigid-body docking. ZDOCK searches for the translation and rotation spaces of two proteins and then ranks each possible pose by ZDOCK score. We used ZDOCK version 3.0.2 for testing and generated 30 docking predictions for each case. As in previous ZDOCK studies [26], we treated the mAb structures and sdAb structures differently. For the mAb structures, we distinguished between heavy and light chains and performed the ‘block’ operation based on the Chothia numbering [38] structure, thereby avoiding antibody framework residues presenting in the predicted interfaces. The range of residues involved in the block is Heavy chain: 6–22, 39–46, 81–91, 106-end; Light chain: 7–20, 38–44, 76–85, 101-end.

#### ClusPro

ClusPro is a protein–protein docking algorithm based on FFT sampling and the PIPER algorithm [10, 39]. The ClusPro online server allows for testing and analysis of unbound receptor and ligand structures by submitting them to the server for docking prediction. By using ‘Antibody Mode’ when submitting unbound structures, it is possible to mask non-CDR regions in the antibody structure during the docking process. This model combines the asymmetric statistical potential of improving antibody–antigen complex prediction, which can improve the performance of antibody–antigen complex structure prediction. The ClusPro (version 2.0) server returns ~30 models for each case.

#### HDOCK

HDOCK is a hybrid docking approach that combines template-based modeling with free docking information [35]. It predicts the binding modes and three-dimensional structures of protein complexes by identifying best-fitting conformations of interacting proteins. The method evaluates model quality by employing diverse scoring functions that take into account aspects like interfacial complementarity and energy minimization. Efficient and rapid docking is guaranteed with HDOCK, which accepts protein sequences or structures as input. We used HDOCK lite v1.1 for testing.

#### AlphaFold-Multimer

AlphaFold-Multimer is able to predict protein complex structures by accepting multi-chain inputs of known stoichiometry. AlphaFold2 can achieve structure prediction accuracy comparable with experiments on single-chain proteins. AlphaFold2 achieves end-to-end prediction from protein sequence to protein structure by combining amino acid sequence, MSA and homologous structure information. AlphaFold-Multimer extends AlphaFold2 to multiple chains to achieve protein complex structure prediction. Taking the amino acid sequence as input, AlphaFold-Multimer generates five models for each case.

#### Comparison of different docking algorithms

**Figure 15:**
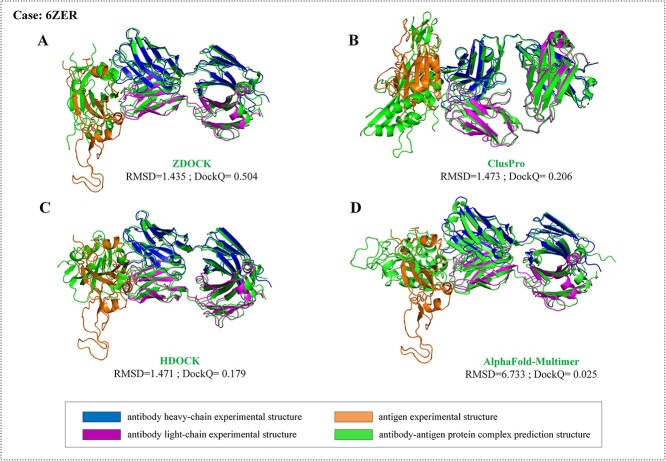
The prediction results by ZDOCK, ClusPro, HDOCK and AlphaFold-Multimer for the case 6ZER. (**A**) Prediction result by ZDOCK. The RMSD and DockQ are 1.435 and 0.504, respectively. (**B**) Prediction result by ClusPro. The RMSD and DockQ are 1.473 and 0.206, respectively. (**C**) Prediction result by HDOCK. The RMSD and DockQ are 1.471 and 0.179, respectively. (**D**) Prediction result by AlphaFold-Multimer. The RMSD and DockQ are 6.733 and 0.025, respectively.

**Figure 16:**
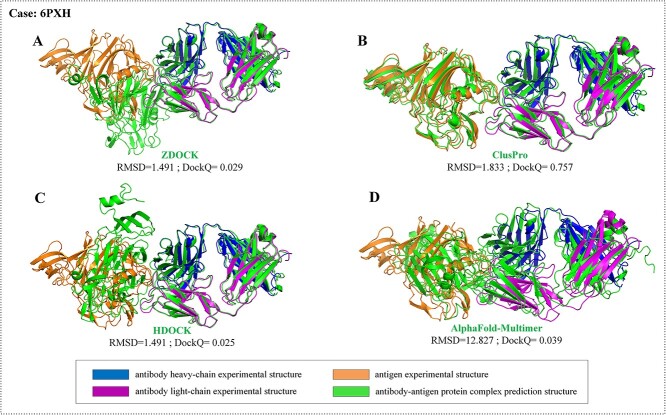
The prediction results when utilizing ZDOCK, ClusPro, HDOCK and AlphaFold-Multimer for case 6PXH. (**A**) Prediction result by ZDOCK. The RMSD and DockQ are 1.491 and 0.029, respectively. (**B**) Prediction result by ClusPro. The RMSD and DockQ are 1.833 and 0.757, respectively. (**C**) Prediction result by HDOCK. The RMSD and DockQ are 1.491 and 0.025, respectively. (**D**) Prediction result by AlphaFold-Multimer. The RMSD and DockQ are 12.827 and 0.039, respectively.

The prediction capabilities of ZDOCK and ClusPro are similar, while the performance of HDOCK is relatively poor. For the top 30, HDOCK produced ~4.5% for Medium accuracy models and 10.7% for Acceptable accuracy models. The reason may be that there is no mode specifically designed for ABAG-docking in HDOCK. The overall success rate of the ClusPro model was higher: 21.4% success for the top 30 and 8.0% for the top 5. In comparison, ZDOCK produced 15.7% success for the top 30 and 6.5% for the top 5. Both ZDOCK and ClusPro are uniquely able to generate Acceptable- or Medium-quality structures for Medium and Difficult cases. For instance, 6ZER is a Medium category case. Only ZDOCK generated models somewhat similar to the native structure, and it achieved Medium-quality models within the top 30. In contrast, other algorithms, including ClusPro and HDOCK, along with AlphaFold-Multimer, were unsuccessful in finding Acceptable conformations within the top 30 (Figure 15A–D). Another example is 6PXH, which is a Medium category case. ClusPro managed to produce Medium-quality models within the top 10, whereas other algorithms failed to find conformations of Acceptable-quality within the top 30 (Figure 16A–D). ZDOCK was able to produce more Medium-quality models: 5.6% success for the top 30 and 0.9% for the top 5. ClusPro produced 4.5% success for the top 30 and did not produce Medium-quality models for the top 5. Both ZDOCK and ClusPro lacked High-quality models, while HDOCK produced one High-quality model on case 6U12.

#### Comparison between AlphaFold-Multimer and docking algorithms

To evaluate the performance of sequence-based protein structure prediction models, we tested AlphaFold-Multimer using the amino acid sequences of antibody–antigen complexes. Compared with docking algorithms, AlphaFold-Multimer had a high overall prediction success rate, ranked the Acceptable or higher quality models higher and generated more Medium- or High-quality models.

AlphaFold-Multimer showed higher overall success rates on the benchmark. The overall success rate of AlphaFold-Multimer was significantly better than that of the docking algorithm for top 5, with success rates of ~9.0% for Medium and higher accuracy models and 15.3% for Acceptable and higher accuracy models. In contrast, ZDOCK produced ~0.9% for Medium and higher accuracy models and 6.5% for Acceptable and higher accuracy models. ClusPro produced 8.0% for Acceptable and higher accuracy models.

AlphaFold-Multimer generated more high-ranking models with Acceptable accuracy or higher accuracy, thereby improving usage efficiency. The accuracy of top 1 generated by AlphaFold-Multimer was not much different from the accuracy of the top 5, with 13.5% success for top 1 and 15.3% success for top 5. For the docking algorithm, ZDOCK produced only 1.9% success for top 1, 6.5% success for top 5 and 15.7% success for top 30; ClusPro produced 3.6% success for top 1, 8.0% success for top 5 and 21.4% success for top 30. This result shows that model ranking has a great impact on docking results, and the performance of the scoring function needs to be improved.

**Figure 17:**
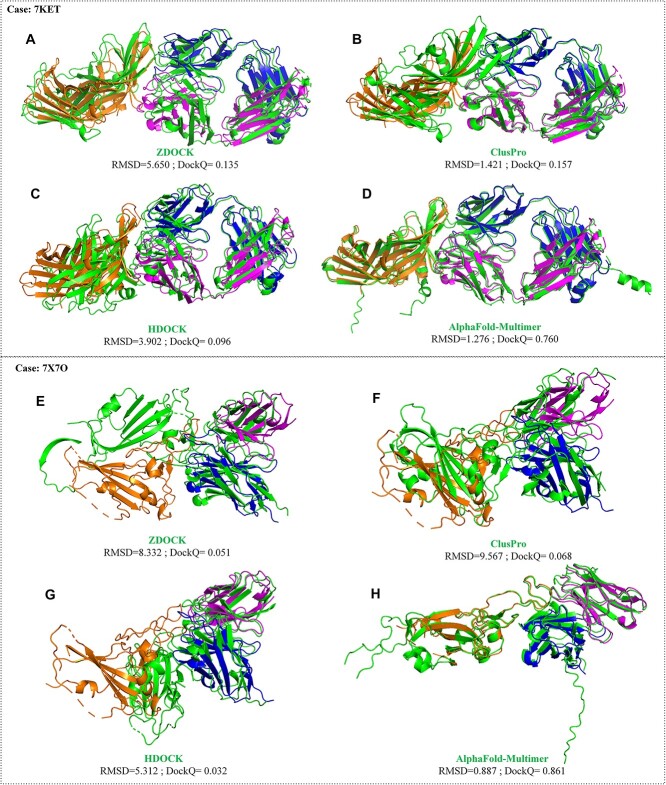
The prediction results when utilizing ZDOCK, ClusPro, HDOCK and AlphaFold-Multimer for cases 7KET and 7X7O. A-D are results for case 7KET . (**A**) Prediction result by ZDOCK. The RMSD and DockQ are 5.650 and 0.135, respectively. (**B**) Prediction result by ClusPro. The RMSD and DockQ are 1.421 and 0.157, respectively. (**C**) Prediction result by HDOCK. The RMSD and DockQ are 3.902 and 0.096, respectively. (**D**) Prediction result by AlphaFold-Multimer. The RMSD and DockQ are 1.276 and 0.760, respectively. E-H are results for case 7X7O. (**E**) Prediction result by ZDOCK. The RMSD and DockQ are 8.332 and 0.051, respectively. (**F**) Prediction result by ClusPro. The RMSD and DockQ are 9.567 and 0.068, respectively. (**G**) Prediction result by HDOCK. The RMSD and DockQ are 5.312 and 0.032, respectively. (**H**) Prediction result by AlphaFold-Multimer. The RMSD and DockQ are 0.887 and 0.861, respectively.

AlphaFold-Multimer produced more Medium accuracy or higher models (9.0% success for the top 5, 8.1% success for top 1), especially High-quality models (2.7% success for top 5, 1.8% success for the top 1). For the docking method, ZDOCK produced less Medium- or High-quality models (0.9% success for top 5) and did not produce High-quality models. ClusPro also lacked High-quality models for top 30 and Medium-quality models for top 5. For instance, 7KET is a Medium category case (Figure 17A–D). Only AlphaFold-Multimer managed to produce models that were closely similar to the native structure, and it achieved Medium-quality models within the top 1. Conversely, other algorithms failed to find Acceptable-quality models even within the top 30. Another example is 7X7O, which is a Rigid category case (Figure 17E–H). AlphaFold-Multimer was capable of generating High-quality models within the top 5, while other algorithms couldn’t find Acceptable-quality models even within the top 30.

In contrast, the docking algorithms produced more diverse models and were better than AlphaFold-Multimer in some cases. For example, ZDOCK and ClusPro produced Medium-quality models on case 6YLA, while AlphaFold-Multimer did not produce Acceptable models.

#### Comparison between different types of cases

Cases under our focus include (i) sdAb and mAb; (ii) two interfaces and (iii) different docking difficulties. We emphasize a comparative analysis from these three perspectives: (i) For sdAb cases, the accuracies of the models predicted by different algorithms are superior to that of mAb cases. Specifically, ZDOCK had a success rate of 28.6% success for the top 30 and 21.4% for the top 5, ClusPro produced 28.6% success for the top 30 and 14.3% success for the top 5, AlphaFold-Multimer achieved 35.7% success for both top 1 and top 5. In contrast, there are some different results for mAb cases. ZDOCK produced 13.8% success for the top 30 and 4.3% success for the top 5. ClusPro produced 20.4% success for the top 30 and 7.1% success for the top 5. AlphaFold-Multimer produced 12.4% success for the top 5. We analyzed that this may be because sdAb cases have fewer conformational changes during binding than mAb cases. (ii) For 14 cases from complexes with two interfaces, the predictions of the algorithms were poor. AlphaFold-Multimer and HDOCK produced Acceptable models for case 7A5S_2, but other algorithms did not produce Acceptable models. We analyzed that the reason may be because the algorithms always try to find a model that minimizes energy, and for cases with two kinds of interfaces, there may be multiple energy minimum points. (iii) For case predictions of three difficulty levels, the success rates of different computational methods are quite different. ZDOCK produced 16.1% success on the Rigid cases, 20.9% success on the Medium cases and 8.8% success on the Difficult cases for the top 30, ClusPro produced 25.8% success on the Rigid cases, 22.2% success on the Medium cases and 16.7% success on the Difficult cases for the top 30, HDOCK produced 12.9% success on the Rigid cases, 11.1% success on the Medium cases and 8.3% success on the Difficult cases for the top 30 and AlphaFold-Multimer produced 25.8% success on the Rigid cases, 13.3% success on the Medium cases and 8.6% success on the Difficult cases for the top 5. For Difficult cases, algorithms generally do not predict them well. Predicting cases with large conformational changes is a huge challenge for docking algorithms, which take unbound structures as input. Although AlphaFold-Multimer does not take the unbound structure as input, compared with the Rigid cases, it does not predict well in cases with large conformational changes before and after binding.

The three different levels of conformational changes affect the degree of docking difficulty, so it is important to use the appropriate docking strategies. For ‘Rigid’ targets, rigid-body docking can yield good results; however, flexibility must be taken into account when docking ‘Difficult’ targets.

## CONCLUSION

Antibody–antigen complex structure datasets play a crucial role in advancing our understanding of the immune system and aiding in the development of therapeutic antibodies. Benchmark datasets provide a wealth of information on the structural details of interactions between antibodies and antigens, offering insights into the molecular mechanisms underlying immune responses. We have constructed a comprehensive and non-redundant benchmark called the ABAG-docking benchmark, which includes 112 diverse cases for ABAG-docking. The benchmark consists of 14 test cases of sdAb and 98 test cases of mAb. According to the conformational changes observed at their interfaces, the 112 targets of the benchmark were grouped into 31 ‘Rigid’, 45 ‘Medium’ and 36 ‘Difficult’ cases. We also recorded different unbound conformations, bound–unbound and unbound–bound cases. A preliminary global docking test on the cases of antibody–antigen complex showed that the ABAG-docking problem remains a challenge and requires the development of more accurate docking algorithms and scoring functions. Antibody–antigen complex prediction is also a very challenging sub-task for protein structure prediction. Docking algorithms and sequence-based structure prediction methods each produce Acceptable models for different cases and can complement each other. The present benchmark is valuable for antibody–antigen protein docking and scoring. The benchmark will be updated annually and is available at https://github.com/Zhaonan99/Antibody-antigen-complex-structure-benchmark-dataset.

In conclusion, the development and analysis of the benchmark dataset for antibody–antigen complexes presented in this study represent a significant step forward in the field of computational biology and immunology. The meticulous curation and categorization of complex structures, based on various criteria, provide researchers with a valuable resource for benchmarking and advancing computational methods. Researchers and scientists can utilize these datasets to explore the diversity of antibody–antigen interactions and design novel antibodies for various applications, including immunotherapy and vaccine development. Furthermore, the introduction of a systematic pipeline for dataset updates ensures its ongoing relevance. As research in the realm of antibody–antigen interactions continues to evolve, this benchmark dataset will empower scientists to develop more accurate and effective tools for antibody–antigen complex prediction and design, ultimately leading to advancements in drug discovery, immunotherapy and disease treatment.

**Key Points**

- Initially, we reviewed the existing publicly available antibody–antigen complex structure benchmark datasets for docking predictions, including Benchmark 4.0, Benchmark 5.0 and Benchmark 5.5.
- We proposed a comprehensive antibody–antigen structure dataset for complex docking prediction, named the ABAG-docking benchmark, which contains various docking difficulty-level cases. Notably, there is a significant increase in Difficult cases, contributing to the development of more effective prediction algorithms capable of handling large conformational changes.
- We presented a novel graph-based redundancy approach to remove redundant data by considering sequence and structure similarities between protein complexes.
- We provided an updated pipeline for the benchmark dataset presented, which can regularly add new non-redundant antibody–antigen complex structures released by experiments.
- Several advanced docking algorithms were evaluated on the ABAG-docking benchmark dataset and compared with end-to-end deep learning models. The results showed that there is still much room for improvement in the antibody–antigen complex structure prediction task.

## Supplementary Material

Supplementary_Material_bbae048

Supplementary_Excel1_ABAG-Docking_Benchmark_Summary_bbae048

Supplementary_Excel2_Unbound_Structure_Id_List_bbae048

## Data Availability

All the processed data of ABAG-docking are available on GitHub: https://github.com/Zhaonan99/Antibody-antigen-complex-structure-benchmark-dataset.

## Author Biographies

**Nan Zhao** is a PhD student in Bioinformatics at Renmin University of China, Beijing, China. Her research studies are focused on protein-protein interactions and non-interactions prediction and protein complex structure prediction.

**Bingqing Han** is a PhD student in Bioinformatics at Renmin University of China, Beijing, China. Her research studies are focused on intrinsically disordered proteins and protein complex structure prediction.

**Cuicui Zhao** is a PhD student in Bioinformatics at Renmin University of China, Beijing, China. Her research studies are focused on biological signal processing, cryo-electron microscope image processing, image classification and denoising.

**Jinbo Xu**'s research lies in the interface of machine learning and AI, computer algorithms and computational biology, i.e., studying AI and computer algorithms to analyze and interpret high volumes of biological data (especially proteomics data) and based upon which building predictive models. He is known for a protein structure prediction program RaptorX, which has been ranked very top in CASPs (Critical Assessment of Structure Prediction) and more importantly, widely used by the broader community. His other work includes machine learning methods, protein de novo sequencing, transcript assembly, protein homology search, protein-protein interaction prediction and functional prediction of variants.

**Xinqi Gong** is a professor in Bioinformatics at Renmin University of China, Beijing, China. His research focuses on mathematical model building, develops computational methods and applies them to study the structure, network and dynamics of multimeric super-large protein interaction complexes; in the direction of machine learning, he uses deep learning and big data methods to design new algorithm frameworks to solve the calculation of biological macromolecules and medical images.
