# Supplementary material for: ABAG-docking benchmark: a non-redundant structure benchmark dataset for antibody–antigen computational docking
Source: Brief Bioinform. 2024 Feb 21;25(2):bbae048. doi: 10.1093/bib/bbae048 (PMC10883643; doi:10.1093/bib/bbae048)
Supplement: Supplementary_Material_bbae048 [file supplementary_material_bbae048.docx]

**Supporting Materials**

**Table S1.** Detailed information on databases and algorithms.

| **Databases and algorithms** | **Description** | **URL** |
| --- | --- | --- |
| Protein Data Bank(1) | The Protein Data Bank (PDB) is a comprehensive and internationally recognized repository that stores three-dimensional structural data of biological macromolecules, providing a valuable resource for researchers in structural biology and related fields. | https://www.rcsb.org |
| SAbDab(2) | SAbDab is a database of all antibody structures in the PDB, each annotated with a large number of attributes including experimental details, antibody nomenclature, affinity data, and sequence annotation. | https://opig.stats.ox.ac.uk/webapps/sabdab-sabpred/sabdab/ |
| SACS(3) | SACS is a list of antibodies extracted from the PDB, containing automatically extracted information. | <http://www.bioinf.org.uk/abs/sacs/> |
| Benchmark 5.0(4) | It describes an updated and integrated version of widely used protein-protein docking and binding affinity benchmarks. | http://zlab.umassmed.edu/benchmark |
| Benchmark 5.5(5) | It describes a non-redundant dataset of antibody-antigen complex structures. | https://zlab.umassmed.edu/benchmark/ |
| ZDOCK | ZDOCK is an algorithm for fast rigid-body docking based on the Fast Fourier Transform by six-dimensional sampling in translational and rotational space. | http://zdock.umassmed.edu/software/ |
| ClusPro(6) | ClusPro is a protein-protein docking algorithm based on FFT sampling and the PIPER algorithm. In particular, there is an option to use "antibody mode" when submitting unbound structures to predict the structure of an antibody-antigen complex, which masks non-CDR regions in the antibody structure during docking. | https://cluspro.bu.edu |
| HDOCK(7) | HDOCK is a computational docking method that combines template-based modeling and free docking information to predict binding modes and three-dimensional structures of protein complexes by finding the best-fitting conformations of interacting proteins. | http://hdock.phys.hust.edu.cn/ |
| PyMOL(8) | PyMOL is a widely used molecular visualization software that allows for the creation and analysis of 3D molecular structures, making it a valuable tool in structural biology and related fields. | https://pymol.org/2/ |

**Table S2.** Antigen lengths of unbound-unbound non-redundant dataset.

| **Complex_id** | **Antigen_chain** | **Chain_length** | **Complex_id** | **Antigen_chain** | **Chain_length** | **Complex_id** | **Antigen_chain** | **Chain_length** |
| --- | --- | --- | --- | --- | --- | --- | --- | --- |
| 5ZUF | A | 297 | 6U54 | B | 118 | 6ZLR | E | 231 |
| 5ZUF | B | 245 | 6UUH | E | 66 | 6ZTR | I | 219 |
| 5ZUF | C | 242 | 6VN0 | A | 475 | 7A5S_1 | A | 1287 |
| 6HER | A | 110 | 6VN0 | B | 153 | 7A5S_2 | B | 1287 |
| 6HHD_1 | A | 108 | 6VO1 | A | 475 | 7C01 | A | 229 |
| 6HHD_2 | C | 107 | 6VO1 | B | 153 | 7CJ2 | B | 362 |
| 6JB8 | B | 129 | 6W4S | F | 605 | 7CQC | A | 192 |
| 6JHT | A | 278 | 6W52 | A | 82 | 7DUO | B | 229 |
| 6JHT | B | 222 | 6W52 | B | 414 | 7EW5_1 | B | 221 |
| 6JHT | C | 246 | 6WIZ | A | 327 | 7EW5_2 | P | 496 |
| 6LGW | E | 409 | 6WIZ | B | 174 | 7JVA | A | 1281 |
| 6OEJ | G | 385 | 6WJ1 | E | 330 | 7KDD | A | 907 |
| 6OFI | G | 355 | 6WJ1 | F | 175 | 7KDD | B | 907 |
| 6ORN | G | 481 | 6X97 | A | 516 | 7KDD | C | 907 |
| 6ORN | C | 153 | 6X97 | B | 153 | 7KET | C | 263 |
| 6OTC | A | 129 | 6XC2 | A | 231 | 7KF0 | V | 116 |
| 6P4B | C | 129 | 6XZF | A | 238 | 7KF1 | V | 116 |
| 6P50 | C | 223 | 6YIO | B | 203 | 7KFW | A | 228 |
| 6PXH | A | 342 | 6YLA | E | 213 | 7L5J | A | 320 |
| 6PZ8 | A | 472 | 6Z3P | A | 297 | 7LO6 | A | 479 |
| 6PZ8 | B | 726 | 6Z3P | B | 254 | 7LO6 | B | 153 |
| 6PZW | A | 429 | 6Z3P | C | 242 | 7LVW | E | 500 |
| 6PZZ | A | 429 | 6Z3P | D | 58 | 7M3L | A | 584 |
| 6Q0O | A | 223 | 6Z3Q | A | 297 | 7M3N | A | 584 |
| 6QB6 | A | 162 | 6Z3Q | B | 254 | 7MMN | C | 72 |
| 6QD7 | C | 322 | 6Z3Q | C | 242 | 7MMN | I | 414 |
| 6QD7 | D | 168 | 6Z3Q | D | 58 | 7MPG | A | 496 |
| 6QFA | B | 341 | 6ZDG_1 | A | 194 | 7ND0 | A | 810 |
| 6QFC | A | 162 | 6ZDG_2 | E | 194 | 7ND0 | B | 392 |
| 6SV2 | A | 113 | 6ZDH | A | 1288 | 7ND0 | C | 344 |
| 6TYS | A | 538 | 6ZER | E | 203 | 7ND0 | D | 245 |
| 6U12 | A | 229 | 6ZFO | E | 194 | 7ND0 | E | 123 |
| **Complex_id** | **Antigen_chain** | **Chain_length** | **Complex_id** | **Antigen_chain** | **Chain_length** | **Complex_id** | **Antigen_chain** | **Chain_length** |
| 7NFD | A | 655 | 7TFO | X | 153 | 8GZ5 | A | 206 |
| 7NFD | B | 655 | 7TYV | A | 259 |  |  |  |
| 7NP1 | A | 232 | 7TYV | a | 164 |  |  |  |
| 7NX3_1 | A | 389 | 7UED | M | 312 |  |  |  |
| 7NX3_2 | F | 389 | 7VNG | D | 148 |  |  |  |
| 7O9W | A | 1280 | 7VYR | R | 262 |  |  |  |
| 7OM4 | A | 630 | 7WO5 | A | 1288 |  |  |  |
| 7OM4 | C | 53 | 7WRV | C | 1205 |  |  |  |
| 7R40 | A | 1275 | 7X7O | B | 215 |  |  |  |
| 7S0E | A | 1271 | 7XXL | B | 204 |  |  |  |
| 7SBG | C | 135 | 7YQX_1 | A | 1270 |  |  |  |
| 7SD3 | A | 481 | 7YQX_1 | B | 1270 |  |  |  |
| 7SD3 | B | 173 | 7YQX_1 | C | 1270 |  |  |  |
| 7SD3 | C | 481 | 7YQX_2 | A | 1270 |  |  |  |
| 7SD3 | D | 173 | 7YQX_2 | B | 1270 |  |  |  |
| 7SD3 | E | 481 | 7YQX_2 | C | 1270 |  |  |  |
| 7SD3 | F | 173 | 7YQZ_1 | A | 1270 |  |  |  |
| 7SGM | A | 146 | 7YQZ_1 | B | 1270 |  |  |  |
| 7SHY | A | 247 | 7YQZ_1 | C | 1270 |  |  |  |
| 7SJN | A | 207 | 7YQZ_2 | A | 1270 |  |  |  |
| 7SJO | A | 235 | 7YQZ_2 | B | 1270 |  |  |  |
| 7SOC | A | 1277 | 7YQZ_2 | C | 1270 |  |  |  |
| 7SU0 | C | 125 | 7Z2M | G | 122 |  |  |  |
| 7SU1 | C | 125 | 7Z2M | I | 122 |  |  |  |
| 7SWN | A | 194 | 7Z4T | I | 153 |  |  |  |
| 7T25 | E | 256 | 7ZF9 | E | 202 |  |  |  |
| 7T73 | E | 504 | 7ZR7 | B | 1285 |  |  |  |
| 7T73 | F | 162 | 8B7W | C | 122 |  |  |  |
| 7T77 | E | 506 | 8F8X | B | 224 |  |  |  |
| 7T77 | F | 162 | 8GV6 | C | 325 |  |  |  |
| 7TEE | C | 862 | 8GV6 | D | 181 |  |  |  |
| 7TEE | D | 883 | 8GV7 | A | 325 |  |  |  |
| 7TFO | A | 481 | 8GV7 | B | 176 |  |  |  |

**Table S3.** Unbound-unbound non-redundant dataset experimental information.

| **Entry ID** | **Resolution (Å)** | **Experimental Method** | **Release Date** | **Entry ID** | **Resolution (Å)** | **Experimental Method** | **Release Date** |
| --- | --- | --- | --- | --- | --- | --- | --- |
| 5ZUF | 6.8 | ELECTRON MICROSCOPY | 2019/12/25 | 6X97 | 3.65 | ELECTRON MICROSCOPY | 2021/12/8 |
| 6HER | 1.199 | X-RAY DIFFRACTION | 2019/12/4 | 6XC2 | 3.112 | X-RAY DIFFRACTION | 2020/7/8 |
| 6HHD | 2.102 | X-RAY DIFFRACTION | 2019/12/11 | 6XZF | 1.8 | X-RAY DIFFRACTION | 2021/2/17 |
| 6JB8 | 1.65 | X-RAY DIFFRACTION | 2019/11/6 | 6YIO | 1.83 | X-RAY DIFFRACTION | 2020/11/11 |
| 6JHT | 3.79 | ELECTRON MICROSCOPY | 2020/3/18 | 6YLA | 2.42 | X-RAY DIFFRACTION | 2020/4/15 |
| 6LGW | 2.9037 | X-RAY DIFFRACTION | 2020/2/19 | 6Z3P | 2.8 | ELECTRON MICROSCOPY | 2020/9/2 |
| 6OEJ | 3.45 | X-RAY DIFFRACTION | 2020/7/15 | 6Z3Q | 2.7 | ELECTRON MICROSCOPY | 2020/9/2 |
| 6OFI | 3.85 | X-RAY DIFFRACTION | 2020/6/24 | 6ZDG | 4.7 | ELECTRON MICROSCOPY | 2020/7/29 |
| 6ORN | 4.05 | ELECTRON MICROSCOPY | 2019/6/12 | 6ZDH | 3.7 | ELECTRON MICROSCOPY | 2020/7/1 |
| 6OTC | 1.7 | X-RAY DIFFRACTION | 2019/6/5 | 6ZER | 3.8 | X-RAY DIFFRACTION | 2020/6/24 |
| 6P4B | 1.9 | X-RAY DIFFRACTION | 2020/5/27 | 6ZFO | 4.4 | ELECTRON MICROSCOPY | 2020/7/8 |
| 6P50 | 2.9 | X-RAY DIFFRACTION | 2019/9/4 | 6ZLR | 3.1 | X-RAY DIFFRACTION | 2020/12/23 |
| 6PXH | 2.3 | X-RAY DIFFRACTION | 2019/9/25 | 6ZTR | 2.1 | X-RAY DIFFRACTION | 2021/5/5 |
| 6PZ8 | 4.19 | ELECTRON MICROSCOPY | 2019/10/9 | 7A5S | 3.9 | ELECTRON MICROSCOPY | 2020/9/16 |
| 6PZW | 3 | ELECTRON MICROSCOPY | 2019/12/4 | 7C01 | 2.88 | X-RAY DIFFRACTION | 2020/5/27 |
| 6PZZ | 3.6 | ELECTRON MICROSCOPY | 2019/12/4 | 7CJ2 | 2.7 | X-RAY DIFFRACTION | 2021/7/14 |
| 6Q0O | 3 | X-RAY DIFFRACTION | 2019/12/18 | 7CQC | 2.5 | X-RAY DIFFRACTION | 2021/5/19 |
| 6QB6 | 2.24 | X-RAY DIFFRACTION | 2019/11/6 | 7DUO | 2.81 | X-RAY DIFFRACTION | 2021/3/10 |
| 6QD7 | 3.1 | ELECTRON MICROSCOPY | 2019/10/2 | 7EW5 | 3.606 | X-RAY DIFFRACTION | 2022/6/1 |
| 6QFA | 2.49 | ELECTRON MICROSCOPY | 2021/8/4 | 7JVA | 3.6 | ELECTRON MICROSCOPY | 2020/10/14 |
| 6QFC | 1.96 | X-RAY DIFFRACTION | 2019/11/6 | 7KDD | 3.5 | ELECTRON MICROSCOPY | 2021/3/17 |
| 6SV2 | 2.3 | X-RAY DIFFRACTION | 2020/7/29 | 7KET | 2 | X-RAY DIFFRACTION | 2021/6/9 |
| 6TYS | 3.5 | ELECTRON MICROSCOPY | 2019/10/9 | 7KF0 | 2.32 | X-RAY DIFFRACTION | 2021/11/10 |
| 6U12 | 1.56 | X-RAY DIFFRACTION | 2020/2/12 | 7KF1 | 2.45 | X-RAY DIFFRACTION | 2021/11/10 |
| 6U54 | 1.6 | X-RAY DIFFRACTION | 2019/11/6 | 7KFW | 2.792 | X-RAY DIFFRACTION | 2020/12/2 |
| 6UUH | 2.7 | X-RAY DIFFRACTION | 2020/9/23 | 7L5J | 3.2 | ELECTRON MICROSCOPY | 2021/4/7 |
| 6VN0 | 4.25 | ELECTRON MICROSCOPY | 2020/6/24 | 7LO6 | 3.9 | ELECTRON MICROSCOPY | 2021/4/14 |
| 6VO1 | 3.88 | ELECTRON MICROSCOPY | 2020/7/1 | 7LVW | 2.1 | X-RAY DIFFRACTION | 2021/3/24 |
| 6W4S | 3.2 | ELECTRON MICROSCOPY | 2020/9/9 | 7M3L | 3.2 | ELECTRON MICROSCOPY | 2021/7/28 |
| 6W52 | 3.74 | X-RAY DIFFRACTION | 2020/11/11 | 7M3N | 2.4 | ELECTRON MICROSCOPY | 2021/7/28 |
| 6WIZ | 4.2 | X-RAY DIFFRACTION | 2020/7/1 | 7MMN | 3.57 | X-RAY DIFFRACTION | 2021/9/8 |
| 6WJ1 | 3.503 | X-RAY DIFFRACTION | 2020/7/1 | 7MPG | 3.4 | ELECTRON MICROSCOPY | 2021/9/8 |
| **Entry ID** | **Resolution (Å)** | **Experimental Method** | **Release Date** | **Entry ID** | **Resolution (Å)** | **Experimental Method** | **Release Date** |
| 7ND0 | 5.2 | ELECTRON MICROSCOPY | 2021/6/2 | 7Z4T | 3.3 | X-RAY DIFFRACTION | 2023/2/8 |
| 7NFD | 3.51 | ELECTRON MICROSCOPY | 2021/4/21 | 7ZF9 | 3.25 | X-RAY DIFFRACTION | 2022/6/1 |
| 7NP1 | 2.8 | X-RAY DIFFRACTION | 2021/11/17 | 7ZR7 | 3.7 | ELECTRON MICROSCOPY | 2022/6/1 |
| 7NX3 | 2.81 | X-RAY DIFFRACTION | 2021/10/27 | 8B7W | 2.85 | X-RAY DIFFRACTION | 2022/12/28 |
| 7O9W | 3.5 | ELECTRON MICROSCOPY | 2022/1/12 | 8F8X | 2.6 | X-RAY DIFFRACTION | 2023/3/29 |
| 7OM4 | 6.05 | X-RAY DIFFRACTION | 2022/3/2 | 8GV6 | 3.4 | X-RAY DIFFRACTION | 2022/12/21 |
| 7R40 | 2.9 | ELECTRON MICROSCOPY | 2022/4/20 | 8GV7 | 2.6 | X-RAY DIFFRACTION | 2022/12/21 |
| 7S0E | 4.9 | ELECTRON MICROSCOPY | 2021/10/6 | 8GZ5 | 1.7 | X-RAY DIFFRACTION | 2022/12/7 |
| 7SBG | 3.34 | X-RAY DIFFRACTION | 2022/8/10 |  |  |  |  |
| 7SD3 | 3.67 | ELECTRON MICROSCOPY | 2022/11/9 |  |  |  |  |
| 7SGM | 2 | X-RAY DIFFRACTION | 2022/2/2 |  |  |  |  |
| 7SHY | 3 | X-RAY DIFFRACTION | 2021/12/15 |  |  |  |  |
| 7SJN | 3.4 | ELECTRON MICROSCOPY | 2022/9/7 |  |  |  |  |
| 7SJO | 3.3 | ELECTRON MICROSCOPY | 2022/9/7 |  |  |  |  |
| 7SOC | 3.3 | ELECTRON MICROSCOPY | 2021/11/17 |  |  |  |  |
| 7SU0 | 2.41 | X-RAY DIFFRACTION | 2022/3/2 |  |  |  |  |
| 7SU1 | 2.53 | X-RAY DIFFRACTION | 2022/3/2 |  |  |  |  |
| 7SWN | 4.3 | ELECTRON MICROSCOPY | 2022/4/27 |  |  |  |  |
| 7T25 | 2.25 | X-RAY DIFFRACTION | 2022/12/14 |  |  |  |  |
| 7T73 | 4 | ELECTRON MICROSCOPY | 2022/9/28 |  |  |  |  |
| 7T77 | 4.75 | ELECTRON MICROSCOPY | 2022/9/28 |  |  |  |  |
| 7TEE | 6.59 | ELECTRON MICROSCOPY | 2022/3/2 |  |  |  |  |
| 7TFO | 4.1 | ELECTRON MICROSCOPY | 2022/1/26 |  |  |  |  |
| 7TYV | 2.8 | ELECTRON MICROSCOPY | 2022/6/1 |  |  |  |  |
| 7UED | 3 | X-RAY DIFFRACTION | 2023/2/1 |  |  |  |  |
| 7VNG | 3.8 | X-RAY DIFFRACTION | 2022/10/26 |  |  |  |  |
| 7VYR | 2.2 | X-RAY DIFFRACTION | 2022/3/2 |  |  |  |  |
| 7WO5 | 3.45 | ELECTRON MICROSCOPY | 2022/7/20 |  |  |  |  |
| 7WRV | 2.47 | ELECTRON MICROSCOPY | 2022/3/23 |  |  |  |  |
| 7X7O | 3.75 | X-RAY DIFFRACTION | 2022/5/25 |  |  |  |  |
| 7YQX | 3.72 | ELECTRON MICROSCOPY | 2022/10/19 |  |  |  |  |
| 7YQZ | 3.84 | ELECTRON MICROSCOPY | 2022/10/19 |  |  |  |  |
| 7Z2M | 1.899 | X-RAY DIFFRACTION | 2023/2/8 |  |  |  |  |

**Table S4.** Antibody CDR loop sequences.

| **ID** | **CDRH1** | **CDRH2** | **CDRH3** | **CDRL1** | **CDRL2** | **CDRL3** |
| --- | --- | --- | --- | --- | --- | --- |
| 5ZUF | AASGFSFSTYGMS | TISGGGGYTY | ARRVTTVAEYYFDY | SASSSVSYIH | YDTSRLAF | QQWSSNYT |
| 6HER | AASGRTFSSYNMG | SITSSGDKSD | ARGLGIYIIRARGGYDH |  |  |  |
| 6HHD | AASGRTFSSYNMG | SDYTDSVKGRFTI | RARGGYDHWGQGTQVTVSS |  |  |  |
| 6JB8 | AASGSTDSIEYMT | ALYTHTGNTY | GATRKYVPVRFALDQSSYDY |  |  |  |
| 6JHT | AASAFTITTYGMS | TITAGGSYTY | ARKVTSVAEYYFDY | SATSGLSYIH | YDTSKLAF | QQWDVNPYT |
| 6LGW | KASGYTFTDYNMD | DISPYYGSTG | ARRNYDGSWFAY | RASQIIGTSIH | KYASESIS | QQSNSWPVT |
| 6OEJ | AASGFTFSSYSMN | SISNTSTYIY | ARANQHFDWLLSLLGGYHYYGMDV | RSSQSLLHSNGYNYLD | YLGSNRAS | MQALQAVG |
| 6OFI | KASGYIFISYFMH | IINPSSGDTR | ARRPGGLERHNWLDP | RASQSISSNLA | YGASTRAT | QQYNNWPAIT |
| 6ORN | SVSGDSMNNYYWT | YISDRESAT | ATARRGQRIYGVVSFGEFFYYYSMDV | GRQALGSRAVQ | YNNQDRPS | HMWDSRSGFSWS |
| 6OTC | AASGFNISYYYIH | SIYPYYGYTS | ARGSSWYGAHAFDY | RASQSVSSAVA | YSASSLYS | QQGYYYAYSLIT |
| 6P4B | SVTGDSITSDHWS | YVSYSGNTF | ANWDGDY | RASQSIGNNLH | KYASQSIS | QQSNSWPYT |
| 6P50 | KASGYTFTSYWMH | EIDPSDSYTN | ARRLYSNSYYYAMDY | KASQDIKKYIA | HYTSTLQP | LQYDNLLT |
| 6PXH | KTSGFTFSSSYIS | WIYAGTGGTE | ARGGSSFAMDY | RASESVDNYGISFMN | HTASNQGS | QQSEEVPLT |
| 6PZ8 | KTSGFTFSSSYIS | WIYAGTGGTE | ARGGSSFAMDY | RASESVDNYGISFMN | HTASNQGS | QQSEEVPLT |
| 6PZW | AASGFTFNNYGMH | VISYDGSNKY | AKDKRFITMILVGPFDY | TGTSSDVGGYNYVS | YDVSNRPS | SSYTSSTTNV |
| 6PZZ | KASGYTFISYGIS | WISAYNGNTN | ARVIPGTAVDYFDY | RASQSISSYLN | YAASSLQS | QQSYSAPFT |
| 6Q0O | AVSGGSISSSQWWS | EISHGVYTN | ARAPPYCSSASCPDDYYYFFLDV | QGDSLRGYYAS | YGKNNRPS | ASRDSSGNHPVV |
| 6QB6 | AASGFTFSSYSMN | SISSSSSYIY | ARQVGATWAFDI | SGSSSNIGSNTVN | YSNNQRPS | AAWDDSLNAWV |
| 6QD7 | EASGFPLRDYAMS | TIGGNDNAAN | AKSVRLSRPSPFDL | RASQSVSTYLA | YEASNRAT | QQRASWPLT |
| 6QFA | AASGHTFNYPIMG | AISWSGGSTS | AAKGRYSGGLYYPTNYDY |  |  |  |
| 6QFC | AASGFTFSSYSMN | SISSSSSYIY | ARQVGATWAFDI | SGSSSNIGSNTVN | YSNNQRPS | AAWDDSLNAWV |
| 6SV2 | KASRNTFTDYNLD | NVYPNNGVTG | ALYYYDVSY | SASSSVSYMH | YDTSKLAS | HQWRSNPYT |
| 6TYS | AASGFTFSSYDMS | MISSGGSYSY | ARQGDYAWFAY | LASQTIGTWLA | YAATSLAD | QQFYSTPFT |
| 6U12 | AASGHTYSTYAMG | RINVGGSSTW | TLHRFANTWSLGTLNV |  |  |  |
| 6U54 | AASGSISDFAAMA | TIFSAGALL | RLYAEAIY |  |  |  |
| 6UUH | KPVGGTFTNFAIH | GRVPVVGIYK | TRWRGCGMCPYDTSSYYNDASDV | RASQNISSSWIA | SAASARAA | QYYGGSFFT |
| 6VN0 | AASEFSFSTHDMH | GINIHGGTY | ARGGKPIYYSGGYPSWYFDL | QASQTIGTNLH | KYSSQSIS | QQTNSFPCT |
| 6VO1 | AVSGGSISGGYGWT | NIYGHSGSTN | ARWSTADFDY | RASQDITNDLA | YYASNLES | QQHNNYPLT |
| 6W4S | SVTGSSITSDYWN | YISYSGSTY | ARQGLRNWYFDV | RASKSVSASAYSYMH | YLASNLES | QHNRELPYT |
| 6W52 | KTSGGTYGTYSIN | AIIPIFGKTN | ARVEDTALDHYFDY | TGTSGDVGTYNYVS | YDVTRRPS | CSYAGTLTWV |
| 6WIZ | AISGDSVSSSSAVWT | RTYYRSKWYDD | ARSSINIFGVFVMAMDV | RASQTVYNSYLA | YGTSTRAT | QQYSTSPRALT |
| 6WJ1 | AISGDSVSSNSVAWN | RTYFRSKWYTD | VRGIIFNWPLGGWSFDL | RASQSVASSYLA | YATINRAA | QQFDSSSMYT |
| **ID** | **CDRH1** | **CDRH2** | **CDRH3** | **CDRL1** | **CDRL2** | **CDRL3** |
| 6X97 | KASGFDFSDNYYIC | CIFTQNVRTY | ARFSDTGPDYGLGNL | QASQRIGSHVS | YGASNLES | QATYDPYTGGSYGAG |
| 6XC2 | AASGLTVSSNYMS | VIYSGGSTF | ARDLDVYGLDV | RASQGISSYLA | YAASTLQS | QQLNSYPPKFT |
| 6XZF | AASGFPVNRYSMR | GMSSAGDRSS | NVNVGFEY |  |  |  |
| 6YIO | KASGGTFSSLAIS | GIIPIFGTAN | ARGGSVSGTLVDFDI | RASQSISSWLA | YKASSLES | QQYNIYPIT |
| 6YLA | KGSGYGFITYWIG | IIYPGDSETR | AGGSGISTPMDV | KSSQSVLYSSINKNYLA | YWASTRES | QQYYSTPYT |
| 6Z3P | KGSGYSFTSYWIG | IIYPGDSDTR | ARLHSSSWFYGMDV | RASQSISSYLN | YAASSLQS | QQSYSTPRT |
| 6Z3Q | TVSGGSISSSSYYWG | SIYYSGSTY | AREITMIAWFDP | RASQGIRNDLG | YAASSLQS | LQDYNYLLT |
| 6ZDG | AASAFTFSSYDMH | KYYADSVKGRFTI | VYYFDYWGQGTLVTVSS | RASQSISSYLN | YAASSLQS | QSYSTLALTF |
| 6ZDH | AASAFTFSSYDMH | VISYDGSNKY | AKDGGKLWVYYFDY | RASQSISSYLN | YAASSLQS | QQSYSTLALT |
| 6ZER | AASAFTFSSYDMH | VISYDGSNKY | AKDGGKLWVYYFDY | RASQSISSYLN | YAASSLQS | QQSYSTLALT |
| 6ZFO | AASAFTFSSYDMH | VISYDGSNKY | AKDGGKLWVYYFDY | RASQSISSYLN | YAASSLQS | QQSYSTLALT |
| 6ZLR | KGSGYGFITYWIG | IIYPGDSETR | AGGSGISTPMDV | KSSQSVLYSSINKNYLA | YWASTRES | QQYYSTPYT |
| 6ZTR | AISGDSVSSQSAAWN | RIYYRSKWYND | ARGEGYGREGFAI | RASQTISNTLA | YAASNLQS | QQYLSWFT |
| 7A5S | KGSGYGFITYWIG | TRYSPSFQGQVTI | TPMDVWGQGTTVTVAS | KSSQSVLYSSINKNYLA | YWASTRES | QYYSTPYTF |
| 7C01 | AASGFTVSSNYMS | VIYSGGSTF | ARVLPMYGDYLDY | RASQSISRYLN | YAASSLQS | QQSYSTPPEYT |
| 7CJ2 | AASGFTFSNYAMS | GISGSGGTTY | AGVGTFDV | RASQTISSWLN | YAASRLQS | QQSYSTPLT |
| 7CQC | AASGFTFSNYGMA | SISAGGDKTY | AKTSRVYFDY | KRSTGNIGSNYVN | YRDDKRPD | HSYSSGIV |
| 7DUO | AVSGFTFNSFAMS | AISGSGGGTY | AKDKILWFGEPVFDY | RASQSVSSYLA | YDASNRAT | QQRSNWPPT |
| 7EW5 | TASGFNIKDYAIH | TEYVPKFQGKATM | RGRFPYWGQGTLVTVSA | KASQNVGTAVA | YFASNRYT | QYSSYPLTF |
| 7JVA | AASGFTFSSYWMN | NIKQDGSEKY | ARVWWLRGSFDY | TGSSGSIASNYVQ | YEDNQRPS | QSYDSSNHVV |
| 7KDD | KASGYSLKDHYMV | WINPQSGGTG | ARDGAKTVSNSGLSLLYYHNRLDA | SGSSSNIGKNYVS | FDNNKRPS | GTPDRSLSVI |
| 7KET | VTSGFTFRSYAMT | SISHSGGSTK | AKDQISYPAASPLDY | RASQSFSSFSLA | YAPSNRAT | QQYGSSPIT |
| 7KF0 | AASGFNIKDTYIH | TRYADSVKGRFTI | YYYMDVWGQGTLVTVSS | RASQDIPRSISGYVA | YWGSYLYS | QQHYTTPPT |
| 7KF1 | AASGFNIKDTYIH | TRYADSVKGRFTI | YYGMDVWGQGTLVTVSS | RASQDIPRSISGYVA | YWGSYLYS | QQHYTTPPT |
| 7KFW | AASGFTVSSNYMS | TYYADSVKGRFTI | RYGLDYWGQGTLVTVSG | RASQGISSYLA | YAASTLQS | QQLNSYPGT |
| 7L5J | AASGFDFSRYWMS | INYTPSLRDKFII | YALDYWGQGASVTVSS | SASSSVSYMH | YSTSNLAS | QQRSSYPFT |
| 7LO6 | KASGDTFIRYSFT | AHYAPHLQGRVTI | EYDNNGFLKHWGQGTLVTVSS | RASESVSSDLA | YGASTRAT | QQYNNWPPRYT |
| 7LVW | AASGQTFSGYVTG | LYYADSVQGRFTI | AAEWYDYWGQGTQVTVSS |  |  |  |
| 7M3L | KASGYTFTNYDMN | TRYNEKFKGKATL | SYSFAYWGQGTLVTVSG | KASQDVNTALA | YSASNRYT | QQHYTTPWT |
| 7M3N | KASGYTFTNYDMN | TRYNEKFKGKATL | SYSFAYWGQGTLVTVSG | KASQDVNTALA | YSASNRYT | QHYTTPWTF |
| 7MMN | AASGFSFSHYAMH | TYYADSVKGRFSI | YYYGMDVWGQGATVTVSS | QASQDIKKYLN | HDASNLET | QYDNLPPLTF |
| 7MPG | AASGFSFSHYAMH | TYYADSVKGRFSI | YYYGMDVWGQGATVTVSS | QASQDIKKYLN | HDASNLET | QYDNLPPLTF |
| **ID** | **CDRH1** | **CDRH2** | **CDRH3** | **CDRL1** | **CDRL2** | **CDRL3** |
| 7ND0 | VASRFTFSNYGMN | IYYAETVKGRFTI | GAFFDYWGQGAMVTVSS | RASEDIHSRLA | YNANSLHT | QYYNYPPYTF |
| 7NFD | TVTGFSITSDYAWN | TTYNPSLRGRISI | GTLDYWGQGTSVTVSS | KASGYILNRLA | SGATSLET | QYWSTPWTF |
| 7NP1 | AASGITVSSNYMS | TYYADSVKGRFTI | VGVTSDYWGQGTLVTVSS | RASQSISRYLN | YAASSLQS | QSYSTLPYTF |
| 7NX3 | KASGYAFSSYWVN | TNYNGKFKGKATL | GSTYDSWGQGTTLTVSS | RASESVDNYGISFMN | YAASNQGS | QSKEVPWTF |
| 7O9W | KASGYSFSNYYIH | TFYNQKFKGKATF | NFYPMDYWGQGTTVTVSS | RSSQSLLHSNGNTYLH | YKVSNRFS | QSTHIPPWTF |
| 7OM4 | AASGRSFSTYAMG | TDYADSVKGRFTI | RNVDYDYWGQGTQVTVSS |  |  |  |
| 7R40 | VASGFTFSSYVMS | TYYADSVKGRFTI | YGSGSFWGQGTLVTVSS | RASQSFHNYLA | FDASNRAT | QRFNWPLTF |
| 7S0E | AASGFTFSSYYMH | TYYADSVKGRFTI | DWGFDVWGQGTLVTVSS | QASQDISNYLN | YDASNLET | QWADWPLTF |
| 7SBG | AASGFTFNTYAMN | TYYADSVKDRFTI | GDYWGQGTSVTVSS | RASGNIHNYLA | YNAKTLAD | HFWSTPYTF |
| 7SD3 | KASGGSFSTYALS | TNYAPRFQGRITI | GKPIGAFAHWGQGTLVTVSS | RASQSVGNNKLA | YGASSRPS | QYGQSLSTF |
| 7SGM | KASGYIFTSYYMY | TNFNEKFKSKATL | NDMDSWGQGTLVTVSS | RASQRVSSSTYSYMH | KYASNLES | HSWEXPPTF |
| 7SHY | AVSGYSITSGYSWN | TNYNPSVKGRITI | HWHFAVWGQGTLVTVSS | RASQSVDYDGDSYMN | YAASYLES | QSHEDPYTF |
| 7SJN | KASGYKFTDSEMH | AAYNQKFKGRATI | YALDYWGQGTLVTV | RASSSVEFIH | SATSNLAS | QWSSAPWTF |
| 7SJO | KASGYKFTDSEMH | AAYNQKFKGRATI | YALDYWGQGTLVTVSS | RASSSVEFIH | SATSNLAS | QWSSAPWTF |
| 7SOC | KASGYPFTSYGIS | TNYAQKFQGRVTM | ESLIGGFDNWGQGTLVTVSS | RASQTVSSTSLA | YGASSRAT | QHDTSLTF |
| 7SU0 | AASGFTFSHYTMH | KYYADSVKGRFTI | GPFDYWGQGTLVTVSS | RASQEVGESELA | YGAFSRAT | QYGSSPWTF |
| 7SU1 | AASGFTFSHYTMH | KYYADSVKGRFTI | GPFDYWGQGTLVTVSS | RASQEVGESELA | YGAFSRAT | QYGSSPWTF |
| 7SWN | KASGFTFTSSAVQ | TNYAQKFQERVTI | CHDAFDIWGQGTMVTVSS | RASQSVSSSYLA | YGASSRAT | QYGSSPWTF |
| 7T25 | KGSGYIFATYWIG | TRYSPSFQGQVTI | YFDLWGRGTLVTVSS | RASQSVSSSYLA | YGASSRAT | QYGSSPLTF |
| 7T73 | AASGFTFSNAWMS | TDYAAPVKGRFTI | GYDDHYYDYYFRDVWGKGTTVTVSS | RASQSVSSSYLA | YGASSRAT | QYGSSFTF |
| 7T77 | AASGFDFSRQGMH | KYHADSVWGRLSI | GYNYYDFYDGYYNYHYMDVWGKGTTVTVSS | QGTSNDVGGYESVS | YDVSKRPS | SLTSTRRRVF |
| 7TEE | AASGFTFSSYTMS | TYYPDTVKGRFTI | SYWYFDVWGAGTTVTVSS | KASQDINKYIA | HYTSSLQP | QYDNLYTF |
| 7TFO | KASGFTFGRYSFT | TNSAKKFQGRVTI | SGYAMDVWGRGALVTVSS | SGSGSNFEYSFVY | YDNYKRPS | SYDSSLTYWVF |
| 7TYV | TASGFNFNKYNMN | IYYADSLKGRFTV | TWSADLWGRGTLVTVSS | RASQSISSSLN | YAAVNLET | QSDTRTF |
| 7UED | KASGYSFTGYTMN | SSYNQKFRGKATL | RGFDYWGSGTPVTVSS | SASSSVSYMH | YDTSKLAS | QWSKHPLTF |
| 7VNG | AASGFNISSSYIH | TYYADSVKGRFTI | AGGGLDYWGQGTLVTVSS | RASQSVSSAVA | YSASSLYS | QGYAWRNTF |
| 7VYR | KASGGTFGNWSIS | ATYAQKFQGRVTI | GGMDVWGQGTTVTVSS | GGNNIGRKSVH | YDDSDRPS | VYDLFSDYVF |
| 7WO5 | AASGFTFSSYWMS | KYYVDSVKGRFTI | GPRDYWGQGTLVTVSS | TRSSGSIASNYVQ | YEDNQRPS | SYDGSNHNVVF |
| 7WRV | KASGGTFSSYAIS | ANYAQKFQGRVTI | WEDVFDIWGQGTMVTVSS | RASQGISSWLA | YDASNLET | QYDNLPLTF |
| 7X7O | KASGFTFSISAVQ | TNYAQKFQERVTI | CSDGFDIWGQGTMVTVSS | RASQSVSSSYLA | YGASSRAT | QYGNSPWTF |
|  |  |  |  |  |  |  |
| **ID** | **CDRH1** | **CDRH2** | **CDRH3** | **CDRL1** | **CDRL2** | **CDRL3** |
| 7YQX | KASGYPFTSYGIS | TNYAQKFQGRVTM | ESLIGGFDNWGQGTLVTVS | RASQTVSSTSLA | YGASSRAT | QHDTSLTF |
| 7YQZ | KASGYPFTSYGIS | TNYAQKFQGRVTM | ESLIGGFDNWGQGTLVTVS | RASQTVSSTSLA | YGASSRAT | QHDTSLTF |
| 7Z2M | KGSGYSFTSYWIG | TRYSPSFQGQVTI | GAFDIWGQGTMVTVSS | RASQSVFSYLA | YDASNRAT | QYFYWGWPF |
| 7Z4T | KGSGYSFTSYWIG | TRYSPSFQGQVTI | DAFDIWGQGTMVTVSS | RASQSVSSYLA | YDASNRAT | QRSNWMFPF |
| 7ZF9 | AASGVTVSSNYMS | TYYADSVKGRFTI | YGIDVWGQGTTVTVSS | RASQGISSYLA | YAASTLQS | QLDSYPPGYTF |
| 7ZR7 | AASGFPFDDYAIH | IGYADSVKGRFTI | GWYYGLDVWGQGATVTVSS | TGTSSDVGGYNYVS | FEVSKRPS | SYAGNKGVF |
| 8B7W | AASGGTFATSPMG | RIYADSVKGRFTI | YYTGDYDSWGQGTLVTVSS |  |  |  |
| 8F8X | AASPGISRYKTMG | TYYADSVKGRFTV | DPYHYYWGQGTQVTVSS |  |  |  |
| 8GV6 | AASGFPFSSYGMH | KYYADSVKGRFTI | IILNGLDVWGQGTTVTVSS | RATQGISSWLA | FGASSLQS | QAHSFPLTF |
| 8GV7 | AASGFPFSSYGMH | KYYADSVKGRFTI | IILNGLDVWGQGTTVTVSS | RATQGISSWLA | FGASSLQS | QAHSFPLTF |
| 8GZ5 | AASGRTSSVYNMA | TLYADSVKGRLTI | ERNYAYWGQGTQVTVSS |  |  |  |

Note: To assess the binding conformational changes at the CDR level, we used the Aho numbering scheme to renumber the antibody chains by the ANARCI software tool. This treatment retained only the renumbered atoms in the heavy and light chain variable domains in single- and double-chain antibodies with residues outside the Aho range. The specific CDR definitions for assessing conformational changes were defined in the AHO numbering as follows:

CDRH1/sdAb CDR1: 24-42

CDRH2/sdAb CDR2: 57-69

CDRH3/sdAb CDR3: 107-138

CDRL1: 24-42

CDRL2: 57-72

CDRL3: 107-138

**Table S5. Additional details for new antibody-antigen test cases. Related to Table S1.**

| **Complex** | **Antigen** | **Antigen Source** | **Antibody** | **Antibody Source** | **Antibody Type** |
| --- | --- | --- | --- | --- | --- |
| 5ZUF | EV71 | Virus | R10 ANTIBODY | Mus musculoides | mAb |
| 6HER | Mouse prion protein | Mus musculus | Nanobody 484 | Camelus dromedarius | Camelid/VHH |
| 6HHD | Mouse Prion Protein | Mus musculus | Nanobody 484 | Camelus dromedarius | Camelid/VHH |
| 6JB8 | hen egg-white lysozyme | Chicken | Nanobody D3-L11 | Camelus dromedarius | Camelid/VHH |
| 6JHT | HAV | Virus | Neutralizing antibody-F9 | Homo sapiens | mAb |
| 6LGW | Rabies virus glycoprotein | Virus | scFv 523-11 | Mus musculus | mAb |
| 6OEJ | HIV-1 CLADE A/E GP120 | Virus | C11 Fab | Homo sapiens | mAb |
| 6OFI | HIV-1 CLADE A/E GP120 | Virus | CH55 Fab | Homo sapiens | mAb |
| 6ORN | Modified BG505 SOSIP-based immunogen RC1 | Virus | 10-1074 antibody Fab | Homo sapiens | mAb |
| 6OTC | Marburg virus VP35 interferon inhibitory domain | Virus | sFab H3 | synthetic construct | mAb |
| 6P4B | hen egg lysozyme variant HEL2x-flex | Chicken | HyHEL10 Fab | Mus musculus | mAb |
| 6P50 | human IL-7Ralpha | Homo sapiens | anti-IL-7R 4A10 Fab | Mus musculus | mAb |
| 6PXH | MERS-CoV S1-NTD | Virus | G2 | Mus musculus | mAb |
| 6PZ8 | MERS S0 trimer | Virus | G2 | Mus musculus | mAb |
| 6PZW | N9 Shanghai2 | Virus | NA-22 fragment antigen binding | Homo sapiens | mAb |
| 6PZZ | N9 Shanghai2 | Virus | NA-80 fragment antibody | Homo sapiens | mAb |
| 6Q0O | influenza hemagglutinin head domain of A/Solomon Islands/3/2006(H1N1) | Virus | H2227 Fab | Homo sapiens | mAb |
| 6QB6 | Mcl1 | Homo sapiens | Antibody fragment | Homo sapiens | mAb |
| 6QD7 | EBOV-GP | Virus | 3T0331 neutralizing antibody | Homo sapiens | mAb |
| 6QFA | beta3K279T GABA(A)R homomer | Homo sapiens | Mb-c7HopQ-Nb25 | Helicobacter pylori | scFv |
| 6QFC | Mcl1 | Homo sapiens | anti-Mcl1 scFv | Homo sapiens | scFv |
| 6SV2 | Human prion protein (PrP) fragment 119-231 (G127V M129 variant) | Homo sapiens | ICSM 18-ANTI-PRP THERAPEUTIC FAB | Mus musculus | mAb |
| 6TYS | the fusion glycoprotein inhibits Nipah virus and Hendra virus infection | Virus | 5B3 antibody | Mus musculus | mAb |
| 6U12 | LRR domain of InlB | Bacterium | VHH R303 C33A/C102A mutant | Camelus dromedarius | Camelid/VHH |
| 6U54 | Zaire ebolavirus Nucleoprotein C-terminal Domain 634-739 | Virus | Anti-Zaire ebolavirus Nucleoprotein Single Domain Antibody Zaire C (ZC) | Lama glama | Camelid/VHH |
| 6UUH | HIV-1 | Bacterium | B11 Fab | Homo sapiens | mAb |
| 6VN0 | BG505 SOSIP.v4.1 | Virus | RM20F Fab | Macaca mulatta | mAb |
| 6VO1 | BG505 SOSIP.v5.2 | Virus | RM20J Fab | Macaca mulatta | mAb |
| 6W4S | apo human ferroportin | Homo sapiens | Fab45D8 | Mus musculus | mAb |
| 6W52 | Prefusion RSV F | Virus | RSB1 Fab | Homo sapiens | mAb |
| 6WIZ | H1 influenza hemagglutinin | Virus | Fab 54-1G05 | Homo sapiens | mAb |
| 6WJ1 | H1 influenza hemagglutinin | Virus | Fab 54-4H03 | Homo sapiens | mAb |
| 6X97 | HIV-1 Env BG505 SOSIP.664 | Virus | monoclonal antibody 11A fragment antigen binding | Oryctolagus cuniculus | mAb |
| 6XC2 | SARS-CoV-2 receptor binding domain | Virus | CC12.1 | Homo sapiens | mAb |
| 6XZF | eGFP | Aequorea victoria (Animal) | Nanobody targeted against eGFP | Lama glama | Camelid/VHH |
| 6YIO | CD25 ECD | Homo sapiens | FAB RG6292 | Homo sapiens | mAb |
| 6YLA | SARS-CoV-2 receptor binding domain | Virus | CR3022 Fab | Homo sapiens | mAb |
| 6Z3P | EV71 | Virus | protective antibody 38-3-11A Fab | Homo sapiens | mAb |
| 6Z3Q | EV71 | Virus | protective antibody 38-1-10A Fab | Homo sapiens | mAb |
| 6ZDG | disordered Spike ectodomain | Virus | EY6A | Homo sapiens | mAb |
| 6ZDH | SARS-CoV-2 Spike glycoprotein | Virus | EY6A | Homo sapiens | mAb |
| 6ZER | SARS-CoV-2 Spike glycoprotein | Virus | EY6A | Homo sapiens | mAb |
| 6ZFO | disordered Spike ectodomain | Virus | EY6A | Homo sapiens | mAb |
| 6ZLR | SARS-CoV-2 Receptor Binding Domain (RBD) | Virus | CR3022 FAB | Homo sapiens | mAb |
| 6ZTR | human P-Cadherin(108-324) | Homo sapiens | CQY684 Fab | Homo sapiens | mAb |
| 7A5S | SARS-CoV-2 spike | Virus | CR3022 Fab | Homo sapiens | mAb |
| 7C01 | SARS-CoV-2 RBD | Virus | CB6 | Homo sapiens | mAb |
| 7CJ2 | human YKL-40 | Homo sapiens | Fab | Homo sapiens | mAb |
| 7CQC | PDZ | Bacterium | Fab of NZ-1 | Rattus norvegicus | mAb |
| 7DUO | CD38 | Homo sapiens | daratumumab fab | Homo sapiens | mAb |
| 7EW5 | HPV6 L1 pentamer | Virus | 13H5 | Mus musculus | mAb |
| 7JVA | SARS-CoV-2 spike | Virus | S2A4 Fab | Homo sapiens | mAb |
| 7KDD | HCMV postfusion gB | Virus | SM5-1 Fab antibody | Homo sapiens | mAb |
| 7KET | meningococcal Factor H binding protein | Bacterium | Immunoglobulin gamma, Fd fragment | Homo sapiens | mAb |
| 7KF0 | VEGF | Homo sapiens | anti-VEGF-A Fab bH1 | Homo sapiens | mAb |
| 7KF1 | VEGF | Homo sapiens | anti-VEGF-A Fab bH1 | Homo sapiens | mAb |
| 7KFW | SARS-CoV-2 | Virus | antibody C1A-B3 Fab | Homo sapiens | mAb |
| 7L5J | Mouse Norovirus Protruding domain | Virus | Anti mouse norovirus mAb A6.2 Fab | Mus musculus | mAb |
| 7LO6 | CD4 mimetic BNM-III-170 | Virus | 17b Fab | Homo sapiens | mAb |
| 7LVW | RSV F | Virus | VHH Cl184 | Lama glama | Camelid/VHH |
| 7M3L | Canine parvovirus | Virus | Fab14 | Mus musculus | mAb |
| 7M3N | Canine parvovirus | Virus | CPV Fab14 | Mus musculus | mAb |
| 7MMN | Prefusion RSV F Glycoprotein | Virus | AM14 Fab | Homo sapiens | mAb |
| 7MPG | Prefusion-stabilized RSV F (DS-Cav1) | Virus | AM14 Fab | Homo sapiens | mAb |
| 7ND0 | the wild-type BAM complex (BamABCDE) | Bacterium | Fab1 | Homo sapiens | mAb |
| 7NFD | ABCG2 | Homo sapiens | 5D3(Fab) | Mus musculus | mAb |
| 7NP1 | SARS-CoV-2 Receptor Binding Domain | Virus | Antibody ION-360 | Homo sapiens | mAb |
| 7NX3 | ALK | Homo sapiens | Fab324 | Mus musculus | mAb |
| 7O9W | Encequidar-bound human P-glycoprotein | Homo sapiens | UIC2 Fab-fragment | Mus musculus | mAb |
| 7OM4 | full extracellular EGFR-EGF complex | Homo sapiens | Nanobody EgB4 | Lama glama | Camelid/VHH |
| 7R40 | SARS-CoV-2 spike glycoprotein | Virus | 87G7 | Homo sapiens | mAb |
| 7S0E | SARS-CoV-2 S1 subunit | Virus | N-612-004 Fab | Homo sapiens | mAb |
| 7SBG | profilin from Hevea brasieliensis (Hev b 8) | Hevea brasiliensis (Plant) | Murine Fab/IgE | Mus musculus | mAb |
| 7SD3 | Cytoplasmic tail deleted HIV-1 Env | Virus | 4E10 Fab | Homo sapiens | mAb |
| 7SGM | CD40L | Homo sapiens | 5c8* Fab | Homo sapiens | mAb |
| 7SHY | IgE-Fc | Homo sapiens | Omalizumab | Homo sapiens | scFv |
| 7SJN | Serine protease HTRA1 | Homo sapiens | Fab15H6.v4 | Homo sapiens | mAb |
| 7SJO | Serine protease HtrA1S328A | Homo sapiens | Fab15H6.v4 | Homo sapiens | mAb |
| 7SOC | SARS-CoV-2 S RBD B.1.617.1 kappa variant S309 Local Refinement | Virus | S309 Fab | Homo sapiens | mAb |
| 7SU0 | CTLA-4 | Homo sapiens | acidic pH-selective Ipilimumab variant Ipi.105 | Homo sapiens | mAb |
| 7SU1 | CTLA-4 | Homo sapiens | acidic pH-selective Ipilimumab variant Ipi.106 | Homo sapiens | mAb |
| 7SWN | SARS-CoV-2 Spike 6P (RBD local reconstruction) | Virus | G32A4 Fab | Homo sapiens | mAb |
| 7T25 | OspA-Fab 319-44 complex structure | Bacterium | 319-44 Fab | Homo sapiens | mAb |
| 7T73 | HIV-1 Envelope ApexGT2.2MUT | Virus | PCT64.LMCA Fab | Homo sapiens | mAb |
| 7T77 | HIV-1 Envelope ApexGT3.N130 | Virus | PG9 Fab | Homo sapiens | mAb |
| 7TEE | GluN1b-2B NMDAR | Rattus norvegicus (Animal) | Fab2 | Mus musculus | mAb |
| 7TFO | HIV-1 Env trimer BG505 SOSIP.664 | Virus | CD4 binding site antibody Ab1573 - Fab | Macaca mulatta | mAb |
| 7TYV | Lassa Virus glycoprotein (Josiah) | Virus | 25.10C Fab | Homo sapiens | mAb |
| 7UED | full length mesothelin | Homo sapiens | MORAb-009 Fab | Mus musculus | mAb |
| 7VNG | human coronavirus 229E spike protein receptor-binding domain | Virus | S11 Fab | Homo sapiens | mAb |
| 7VYR | SARS-CoV-2 Spike RBD | Virus | D27 | Homo sapiens | mAb |
| 7WO5 | SARS-CoV-2 Spike | Virus | mAb15 | Homo sapiens | mAb |
| 7WRV | SARS-CoV-2 Omicron Variant S | Virus | JMB2002 Fab | Mus musculus | mAb |
| 7X7O | SARS-CoV-2 spike RBD | Virus | UT28K Fab | Homo sapiens | mAb |
| 7YQX | SARS-CoV-2 BA.2.75 S Trimer | Virus | S309 | Homo sapiens | mAb |
| 7YQZ | SARS-CoV-2 BA.2.75 S Trimer | Virus | S309 | Homo sapiens | mAb |
| 7Z2M | human IL-17A | Homo sapiens | 11.003 Fab | Homo sapiens | mAb |
| 7Z4T | HUMAN INTERLEUKIN-1 BETA | Homo sapiens | AAL160 Fab | Homo sapiens | mAb |
| 7ZF9 | COVOX-150 Fab (P21) | Virus | COVOX-150 | Homo sapiens | mAb |
| 7ZR7 | SARS-COV-2 BETA SPIKE GLYCOPROTEIN | Virus | Omi-42 | Homo sapiens | mAb |
| 8B7W | IL-17A | Homo sapiens | anti-IL-17A-76 | Lama glama | Camelid/VHH |
| 8F8X | the afucosylated human IgG1 fragment crystal form II | Homo sapiens | Nb.X0 | Camelidae mixed library | Camelid/VHH |
| 8GV6 | influenza hemagglutinin H14 | Virus | PN-SIA28 | Homo sapiens | mAb |
| 8GV7 | influenza hemagglutinin H18 | Virus | PN-SIA28 | Homo sapiens | mAb |
| 8GZ5 | SARS-CoV-2 Alpha variant spike receptor-binding domain | Virus | Nanobody P17 | Vicugna pacos | Camelid/VHH |

**Reference**

1. Burley, S.K., Bhikadiya, C., Bi, C., Bittrich, S., Chen, L., Crichlow, G.V., Christie, C.H., Dalenberg, K., Di Costanzo, L. and Duarte, J.M.J.N.a.r. (2021) RCSB Protein Data Bank: powerful new tools for exploring 3D structures of biological macromolecules for basic and applied research and education in fundamental biology, biomedicine, biotechnology, bioengineering and energy sciences. **49**, D437-D451.

2. Dunbar, J., Krawczyk, K., Leem, J., Baker, T., Fuchs, A., Georges, G., Shi, J. and Deane, C.M.J.N.a.r. (2014) SAbDab: the structural antibody database. **42**, D1140-D1146.

3. Allcorn, L.C. and Martin, A.C.J.B. (2002) SACS—self-maintaining database of antibody crystal structure information. **18**, 175-181.

4. Vreven, T., Moal, I.H., Vangone, A., Pierce, B.G., Kastritis, P.L., Torchala, M., Chaleil, R.A.G., Jiménez-García, B., Bates, P.A., Fernández-Recio, J. *et al.* (2015) Updates to the Integrated Protein-Protein Interaction Benchmarks: Docking Benchmark Version 5 and Affinity Benchmark Version 2. **427 19**, 3031-3041.

5. Guest, J.D., Vreven, T., Zhou, J., Moal, I., Jeliazkov, J.R., Gray, J.J., Weng, Z. and Pierce, B.G.J.S. (2021) An expanded benchmark for antibody-antigen docking and affinity prediction reveals insights into antibody recognition determinants. **29**, 606-621. e605.

6. Brenke, R., Hall, D.R., Chuang, G.-Y., Comeau, S.R., Bohnuud, T., Beglov, D., Schueler-Furman, O., Vajda, S. and Kozakov, D.J.B. (2012) Application of asymmetric statistical potentials to antibody–protein docking. **28**, 2608-2614.

7. Yan, Y., Tao, H., He, J. and Huang, S.-Y.J.N.p. (2020) The HDOCK server for integrated protein–protein docking. **15**, 1829-1852.

8. DeLano, W.L.J.C.N.P.C. (2002) Pymol: An open-source molecular graphics tool. **40**, 82-92.
